# Mouse Models of Frequently Mutated Genes in Acute Myeloid Leukemia

**DOI:** 10.3390/cancers13246192

**Published:** 2021-12-08

**Authors:** Sagarajit Mohanty, Michael Heuser

**Affiliations:** 1 Cancer Biology and Genetics, Memorial Sloan-Kettering Cancer Center, New York, NY 10065, USA; 2 Department of Hematology, Hemostasis, Oncology and Stem Cell Transplantation, Hannover Medical School, 30625 Hannover, Germany

**Keywords:** AML, synergy, leukemia, mutations, transgenic mice, mouse models

## Abstract

**Simple Summary:**

Acute myeloid leukemia is a genetically heterogeneous disease and shows variable treatment outcomes. Genetic profiling has revealed different driver mutations in AML patients. Therefore, it is important to understand the biological impact of these mutations in leukemia transformation. In this review, we discuss the individual and synergistic effects of these mutations in the pathogenesis of leukemia based on the available evidence from mouse models.

**Abstract:**

Acute myeloid leukemia is a clinically and biologically heterogeneous blood cancer with variable prognosis and response to conventional therapies. Comprehensive sequencing enabled the discovery of recurrent mutations and chromosomal aberrations in AML. Mouse models are essential to study the biological function of these genes and to identify relevant drug targets. This comprehensive review describes the evidence currently available from mouse models for the leukemogenic function of mutations in seven functional gene groups: cell signaling genes, epigenetic modifier genes, nucleophosmin 1 (*NPM1*), transcription factors, tumor suppressors, spliceosome genes, and cohesin complex genes. Additionally, we provide a synergy map of frequently cooperating mutations in AML development and correlate prognosis of these mutations with leukemogenicity in mouse models to better understand the co-dependence of mutations in AML.

## 1. Introduction

Acute myeloid leukemia (AML) is characterized by the uncontrolled proliferation of leukemic stem cells and results in cytopenia in peripheral blood [1,2]. The 5-year overall survival rate is 30–35% for AML patients up to the age of 60 years [3], underscoring the need for a better understanding of the heterogeneity of AML to develop novel treatment strategies for patients who respond poorly to currently available therapies. Deep sequencing enabled the discovery of driver mutations in primary and relapsed AML patients [4]. Papaemmanuil et al. identified more than 5000 driver mutations across 76 genes in 1540 AML patients [2]. These major driver genes can be clustered into 7 functional groups: cell signaling genes, epigenetic modifier genes, nucleophosmin 1 (*NPM1*), transcription factors, tumor suppressors, spliceosome genes, and cohesin genes [5]. Identification of these key molecular abnormalities improved prognosis, prediction of treatment outcomes, and measurable residual disease (MRD) monitoring [6,7]. Understanding the pathophysiology of these mutations in disease development will be useful to develop more potent targeted therapeutic options for AML patients.

Genetically engineered mouse models have been developed to study AML progression in vivo. These include induced AML models (by chemicals, viral infection, or irradiation), transgenic mouse models, and patient-derived xenograft (PDX) models [8]. Here, we summarize the biological effects of different AML mutations in overexpression, knockin, or knockout mouse models. 

In this review, we discuss the in vivo oncogenic potential of the most frequently mutated genes in AML patients. Further, this review highlights the biological relevance of clinically mutually exclusive and co-occurring mutations.

## 2. Mouse Models of Genes Involved in Cell Signaling Pathways in Myeloid Malignancies

The signal transduction gene set is the most often mutated gene set in AML. These genes are involved in transducing signals from the cell membrane to the nucleus. This results in the target gene expression of the downstream pathway. In this way, it controls important cellular events such as proliferation, apoptosis, and differentiation.

### 2.1. FLT3

**Figure 1:**
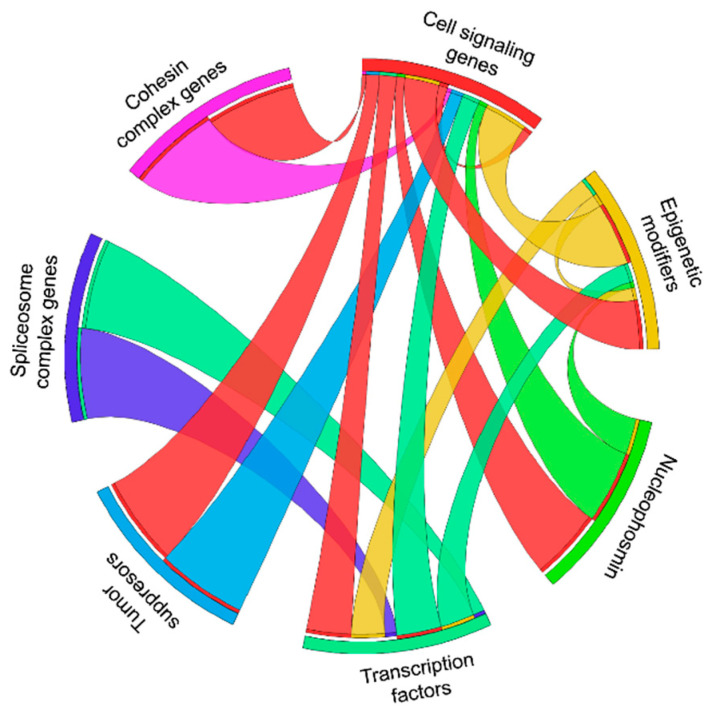
Synergistic AML mouse models by the cooperation of mutations in different functional classes of genes.

The FMS-like tyrosine kinase 3 (*FLT3*) gene encodes a tyrosine kinase receptor, which is activated through the binding of the FLT3 ligand. Two main types of mutations occur in the FLT3 protein. It is either a missense mutation in the tyrosine kinase domain (TKD) or an internal tandem duplication (ITD) mutation in the juxtamembrane domain [9]. Both of these mutations lead to constitutive activation of the FLT3 protein that activates downstream signaling without binding of the ligand. Approximately 30% of AML patients carry *FLT3* mutations. The most common mutation is the ITD mutation which is found in 25% of AML patients [10]. Further, patients with *FLT3-ITD* mutations with a high allelic ratio show poor overall survival [11]. In order to understand the role of Flt3 in leukemogenesis, different mouse models have been established. *Flt3^−/−^* mice are viable but show a deficiency in lymphoid progenitors (Table 1) [12]. Mice receiving a transplant of FLT3-ITD overexpressing bone marrow cells develop a myeloproliferative disorder (MPD)/ myeloproliferative neoplasm (MPN) (Table 1; Supplementary Figure S1) [13,14]. On the contrary, FLT3-TKD mutant mice develop an oligoclonal lymphoid disorder and show long latency of disease compared to FLT3-ITD mice (Table 1; Supplementary Figure S1) [14]. Another mouse model expressing FLT3-ITD under the hematopoietic specific vav promoter shows MPNs and B- or T-lymphoid disorders [15]. The difference in disease phenotypes from the same FLT3-ITD mutation is likely due to its expression from different promoters. Heterozygous and homozygous FLT3-ITD knockin mice develop a myeloproliferative disorder resembling chronic myelomonocytic leukemia (CMML) (Table 1; Supplementary Figure S2) [16]. Another group has also demonstrated that FLT3^wt/ITD^ mice die of fatal MPNs (Table 1; Supplementary Figure S2) [17]. On the other hand, Bailey et al. have shown that FLT3-D835Y knockin mice (resembling FLT3-TKD) develop a less aggressive disease and survive longer compared to FLT3-ITD mice [18]. Both overexpression and knockin mouse models show that TKD mutations develop a disease with longer latency compared to ITD mutations, providing an explanation why the ITD mutation confers an inferior prognosis compared to TKD mutations in AML patients. The stronger disease phenotype in the ITD mice may be due to stronger FLT3 signaling by the ITD mutation compared to the TKD mutation. Additionally, FLT3-D835Y mice develop a broader variability of disease phenotypes (MPNs, lymphomas, histocytic sarcomas, and hemangiosarcomas) compared to FLT3-ITD mice. The above models suggest that the ITD mutation induces stronger myeloid-specific signaling compared to the TKD mutation. Further, *FLT3* mutations alone are not sufficient to induce AML and need additional cooperating mutations. Collaboration mouse models show cooperation of *FLT3* mutations with other mutated genes such as *SMC3* [19], *RUNX1* [20], *NPM1* [21], *DNMT3A* [22,23], *IDH2* [24,25], *WT1* [26,27], *TET2* [28], *SETBP1* [29] and *CUX1* (Table 2; Supplementary Figure S3) [30]. *FLT3* mutations also cooperate with different fusion genes such as *NUP98-NSD1* [31], *NUP98-HOXD13* [32], *KMT2A-AF9* [33] and *RUNX1-RUNX1T1* [34] to develop AML in mice. This illustrates that *FLT3* shows the broadest collaboration with genes from all functional subgroups mutated in AML patients except genes of the splicing complex (Figure 1).

### 2.2. KIT

KIT (CD117) is a transmembrane tyrosine kinase receptor that is activated by binding its ligand SCF [137]. Most common KIT mutations occur in exons 8 and 17 [138,139]. Mutations in both exons lead to the constitutive activation of KIT without the ligand. c-Kit^−/−^ mice show postnatal death with impairment in hematopoiesis (Table 1) [37]. Overexpression of the KIT D816V mutation induces MPNs in mice (Table 1; Supplementary Figure S1) [35,36]. It has been shown that the KIT mutation at exon 17 is a negative prognostic factor in RUNX1-RUNX1T1/AML1-ETO/RUNX1-ETO AML patients [140]. In a cooperation study, c-Kit (D814V) or c-Kit (T417IΔ418–419) induces AML in mice when coexpressed with the RUNX1-RUNX1T1 fusion [141]. Further, KIT D816 (at exon 17) is a hot spot mutation in inv(16) AML, which is characterized by the CBFbeta-MYH11 fusion gene [142]. The cooperation of these two alterations (KIT D816 and CBFbeta-MYH11) also shows synergistic leukemia development in mice [143].

### 2.3. KRAS

RAS family member genes (*NRAS*, *KRAS*, and *HRAS*) are frequently mutated genes in cancers [144]. Proteins encoded by these genes transmit the transcriptional signal from the cell surface to the nucleus [145]. Oncogenic mutations in these genes activate constitutive RAS signaling in the cell. *NRAS* and *KRAS* mutations are frequently detected in AML patients but *HRAS* mutations rarely appear in AML patients [146]. Approximately five percent of AML patients display mutations in the KRAS gene [144,146]. Homozygous deletion of *Kras* results in embryonic lethality (Table 1) [41,42]. Reconstituted mice with bone marrow (BM) progenitor cells that express KRASG12D do not induce any disease (Table 1; Supplementary Figure S1) [38]. However, conditional knock-in of the Kras oncogene leads to the development of MPNs in mice (Table 1; Supplementary Figure S2) [39,40]. These findings support the idea that KRAS mutations are not sufficient for AML development. Therefore, many synergistic mouse models of KRAS mutations with other oncogenes have been established. Co-expression of KRAS mutations with other mutations such as DNMT3A [123], TP53 [124], NF1 [125], and BCOR [126] induces AML in mice (Table 2; Supplementary Figure S3). The KRAS mutation also induces AML in mice when combined with fusion oncogenes such as AML1-ETO [38].

### 2.4. NRAS

*NRAS* mutations account for approximately 10% of AML patients [147]. *NRAS* mutations often appear in codons 12, 13, or 61 [147,148]. Homozygous *Nras* knockout mice show normal growth and no hematological defects (Table 1) [48]. So, unlike Kras, Nras is dispensable for embryonic development. The retroviral overexpression mouse model of NRASG12D does not show any hematological disease development (Table 1; Supplementary Figure S1) [38,43]. However, another study has demonstrated that NRASG12D causes CMML and AML-like disease in mice [44]. Although they have used the MSCV promoter to express the NRAS oncogene, the major discrepancy in the results may be due to the use of a different strain of mice and a high titer of the virus. Moreover, the expression of NRASG12D under the myeloid-specific hMRP8 promoter causes abnormal hyperkeratotic skin lesions in mice [45]. Transgenic Mice expressing oncogenic Nras under the control of the Moloney murine leukemia virus (Mo-MuLV) LTR develop myeloproliferative disorders [46]. This study also observed Nras induced apoptosis in the bone marrow of some mice. Heterozygous NrasG12D knockin mice develop MPNs (Table 1; Supplementary Figure S2) [149]. Secondary transplant recipients from *Nras*^G12D/+^ bone marrow cells develop a CMML-like disease [150]. Homozygous NRASG12D mice die from severe myeloproliferative disease [47]. It suggests that biallelic *NRAS* mutations provide a stronger oncogenic signal. Inactivation of *P53* collaborates with the NRASG12D mutation to induce a highly penetrant AML in vivo (Table 2; Supplementary Figure S3) [127]. Previous studies have shown that overexpression of the *Ras* oncogene causes p53 accumulated cell senescence, which can be overcome by *P53* inactivation [151]. However, the endogenous expression of the *Ras* oncogene does not cause cell senescence [152], indicating involvement of an alternate mechanism for this oncogenic synergy. In contrast to *Nras*^G12D/+^ alone or *p53*^−/−^ alone, cooperation of these mutations induces quiescence in megakaryocyte-erythroid progenitors (MEPs) that is sufficient to drive AML development in mice (Table 2; Supplementary Figure S3) [127]. Similarly, cooperation of the NrasG12D mutation with Dnmt3aR878H/DNMT3AR882H activates the Myc pathway and induces AML in mice (Table 2; Supplementary Figure S3) [128,132]. *NRAS* mutations also show synergistic AML development with mutations in other genes such as *NPM1* [129], *EZH2* [130], and *IDH2* [25] (Table 2; Supplementary Figure S3). Cooperation AML models of oncogenic NRAS has been also established with fusion genes such as NUP98 fusions (NUP98-NSD1, NUP98-JARID1A, and NUP98-DDX10) [43,153], KMT2A-AF9 [154], KMT2A-ENL [155], and RUNX1-RUNX1T1 [155].

### 2.5. NF1

The neurofibromin 1 gene (*NF1*) encodes a RAS GTPase activating protein that modifies active RAS-GTP into its inactive RAS-GDP and suppresses the RAS pathway [156]. Therefore, deletion of this gene or inactivating mutations in this gene triggers the RAS-MAPK signaling pathway. Almost five percent of adult de novo AML patients carry mutations in the *NF1* gene [157]. Homozygous deletion of *Nf1* in embryonic stem cells results in embryonic lethality (Table 1) [49]. On the other hand, *Nf1* heterozygous deletion in embryonic stem cells predisposes mice to develop various tumors including leukemia [50]. Somatic deletion of *Nf1* induces a myeloproliferative disorder in mice that models juvenile myelomonocytic leukemia (JMML) (Table 1) [158]. Another study explains that GM-CSF signaling is indispensable for *Nf1^−/−^* induced MPN development, indicating therapeutic benefit by targeting GM-CSF signaling in *NF1* mutated myeloid disorders [159]. Collaboration of *Nf1* inactivation and the KRASG12D mutation induces AML in mice (Table 2; Supplementary Figure S3) [125]. However, it is unclear whether the synergy is due to hyperactivated RAS signaling or activation of a non-RAS pathway by *Nf1* deficiency. Further, concurrent haploinsufficiency of both *Nf1* and *Asxl1* induces AML in mice [131]. The above-mentioned mouse models of *Nf1* suggest that the *Nf1* mutation is a loss of function mutation and can induce AML in collaboration with other mutations.

### 2.6. PTPN11

The *PTPN11* gene encodes Src homology region 2 (SH2)-containing protein tyrosine phosphatase-2 (SHP2) that regulates the RAS pathway [160]. Germline mutations in the *PTPN11* cause Noonan syndrome and somatic mutations occur in leukemia patients [161]. Mutations in Q510, A72, E76, and G503 are hotspot PTPN11 mutations across different cancers [162]. *PTPN11* mutations frequently occur in JMML patients (around 35%) but are less common in AML patients [163]. Homozygous *Ptpn11* null mice die at the embryonic stage (Table 1) [54]. Knockout of *Ptpn11* in murine hematopoietic cells causes the death of mice due to bone marrow aplasia, indicating its significance for the survival of HSCs [164]. This study also shows that constitutive expression of Kras can rescue *Ptpn11*^Δ/Δ^ HSCs and myeloid progenitor cells that indicate the presence of Kras downstream of Ptpn11 in HSCs [164]. Retrovirally overexpressed models of Ptpn11 mutants induce a JMML-like disease in mice (Table 1; Supplementary Figure S1) [51]. Knockin mice expressing Ptpn11D61Y show fatal MPNs [52]. Similarly, mice carrying the Ptpn11E76K mutation developed MPNs (Table 1; Supplementary Figure S2) [53,165] and later few mice develop acute leukemia. Further, knock-in of the Ptpn11E76K mutation in myeloid and lymphoid progenitors causes AML and acute lymphocytic leukemia (ALL), respectively [166]. In vivo mouse models also show cooperation of PTPN11 mutations with KMT2A fusions such as KMT2A-MLLT3 [167] or KMT2A-MLLT10 [168] in leukemia development. In conclusion, most of the PTPN11 mutations are gain of function mutations and show oncogenic activity in vivo.

## 3. Mouse Models of Epigenetic Modifier Genes in Myeloid Malignancies

### 3.1. DNMT3A

DNA methylation is one of the important epigenetic control mechanisms in both normal development and cancer [169]. DNA methylation usually refers to the conversion of cytosine to 5’ methyl cytosine in CpG islands, which is orchestrated by DNA methyltransferases (DNMTs) [170]. *DNMT3A* mutations are reported in approximately 20% of AML patients and most commonly affect amino acid R882 [171]. *Dnmt3a^−/−^* mice are not embryonic lethal but die at 4 weeks of age (Table 1) [57]. Hematopoietic-specific conditional Dnmt3a knockout mice develop a myelodysplastic syndrome (MDS) or MPN phenotype in mice [58]. In this study, loss of *Dnmt3a* shows unaltered homing to BM but a selective increase in liver homing compared to *Dnmt3a* wild-type bone marrow cells [58]. It suggests that alterations in liver homing play a role in the development of hematopoietic neoplasms. Mice transplanted with *Dnmt3a* knockout HSCs in a competitive microenvironment do not develop any disease [172] but develop a broad spectrum of hematopoietic malignancies in a non-competitive environment (lethally irradiated mice) [173]. Retroviral overexpression of the hotspot *DNMT3A-Arg882His* (*R882H*) mutation induces CMML in mice (Table 1; Supplementary Figure S1) [55]. Further, knockin of the *Dnmt3a* R878H mutation drives AML development in mice with activation of the mTOR pathway (Table 1; Supplementary Figure S2) [56]. Mutations in *Dnamt3a* or loss of *Dnamt3a* synergize with other alterations such as *Kras (G12D/+)* [123], *FLT3-ITD* [22,23], *Npm1^cA/+^* [134], *Bcor^−/−^* [135] and *IDH2* neomorphic mutations [133] to develop AML in mice (Table 2; Supplementary Figure S3). In an *Flt3-ITD/DNMT3A* cooperation AML model, haploinsufficiency of *Dnmt3a* hypomethylates genes such as *Gata3* that causes the transformation of *FLT3-ITD*-induced MPNs to AML. In addition, genes involved in Wnt signaling such as *Cxxc5* and *Emilin2* are also dysregulated by loss of Dnmt3a, but the correlation with its downstream target c-Myc is not established [22]. Another study has shown that the dosage of *Dnmt3a* determines myeloid or lymphoid transformation. FLT3-ITD overexpression in homozygous *Dnamt3a* knockout or heterozygous *Dnmt3a* knockout cells causes T-ALL or AML in mice, respectively [174]. This suggests that the heterozygous *Dnmt3a* knockout predisposes mice to myeloid malignancy, while the homozygous Dnamt3a knockout is more prone to lymphoid malignancies. Celik et al. found that secondary transplantation of primary MDS BM cells from *Dnmt3a* knockout mice progress to AML by acquiring *C-kit* mutations [175]. Another study has shown that the Ptpn11D61Y mutation cooperates with *Dnmt3a^−/+^* to induce rapid myeloproliferative neoplasms in mice [176].

### 3.2. TET2

Ten eleven translocation 2 (TET2) belongs to the TET family protein that converts 5-methylcytosine (5-mC) to 5-hydroxymethylcytosine (5-hmC), an epigenetic modification important for the regulation of transcription [177,178,179]. Loss of function genomic alterations of *TET2* is implicated in 10–20% of AML patients [180,181]. Inactivation of *TET2* in both murine bone marrow (BM) hematopoietic stem/progenitor cells (HSPCs) and human cord blood CD34+ cells show unbalanced myeloid directed differentiation [178,182]. Further, heterozygous loss of *Tet2* initiates aberrant hematopoiesis in vivo [61] and the homozygous loss leads to a wide spectrum of myeloid malignancies including MDS, CMML, and sarcoma (Table 1) [59,60]. Several studies have explored the cooperative effect of *TET2* loss and mutation/ deletion in other sets of genes. Loss of *TET2* cooperates with the *FLT3-ITD* mutation (Table 2; Supplementary Figure S3) [28] or the *TET3* deletion [183,184] to induce AML in mice. Hypermethylation of Gata2 is observed as a synergistic effect of loss of *Tet2* and *Flt3-ITD* mutation that promotes AML, and this can be reversed by restoration of Gata2 [28]. The double knockout of *Tet2* and *Tet3* causes DNA damage and impaired DNA repair compared to the single loss of Tet genes [183]. Apart from *FLT3-ITD*, loss of *TET2* synergizes with other mutations in signal transduction genes such as *NRASG12D* to induce a fully penetrant CMML phenotype by combined suppression of negative regulators of the RAS pathway [185]. Loss of *TET2* with loss of *EZH2* induces an aggressive MDS/MPN phenotype [186]. *TET2* also cooperates with loss of the transcription factor *BCOR* [94] or the *Sf3b1K700E* mutation in developing a myeloid disease in mice [114]. Tara et al. reported distinct DNA hypermethylation patterns as a synergistic result of combined loss of Tet2 and Bcor [94]. Collectively, loss of *TET2* cooperates with genes of most functional classes to induce aberrant hematopoiesis in vivo.

### 3.3. IDH1 and IDH2

Isocitrate dehydrogenases 1 and 2 (IDH1 and IDH2) convert isocitrate to α-ketoglutarate in the TCA cycle. Mutations in these genes allow the formation of the oncometabolite R-2-hydroxyglutarate from α-ketoglutarate [62,187]. Approximately 20% of AML patients show mutations in *IDH1* or *IDH2* [188]. R-2-hydroxyglutarate inhibits TET2 and shows a global DNA hypermethylation in IDH mutant patients [189,190]. *Idh1*^−/−^ or *Idh2*^−/−^ mice are normal and viable (Table 1) [64,67]. Knockin of the IDH1 (R132H) mutation in the hematopoietic compartment causes anemia, splenomegaly, and extramedullary hematopoiesis in mice with altered DNA and histone methylation profiles (Table 1; Supplementary Figure S2) [63]. However, retroviral overexpression of the *IDH1R132C* mutation does not induce any hematopoietic disease (Table 1; Supplementary Figure S1) [62]. The IDH1 mutation combined with Hoxa9 overexpression leads to AML development in mice [62]. A recent study shows that R-2HG aggravates doxorubicin-mediated cardiotoxicity and increases the risk of cardiac dysfunction in mutant IDH patients [191,192]. IDH2R140Q transgenic mice are characterized by extramedullary hematopoiesis, splenomegaly, and expansion of HSPCs [24]. Idh2R140Q knockin mice show higher 2HG levels but do not develop any hematological disease [65,66]. IDH2 mutations cooperate with mutations in other genes such as Dnamt3a^−/−^ [133], *NPM*c+ [193], NrasG12D [25], and Flt3-ITD [24] to induce leukemia in mice (Table 2; Supplementary Figure S3). A synergistic mouse model of *Dnmt3a^−/−^* and Idh2^R140Q^ shows an increase in methylation of histone H3 lysine residues and a decrease in histone H3 lysine acetylation that contributes to leukemia development [133]. Further, it has been shown that these mice are sensitive to histone deacetylase inhibitor treatment [133]. *NPM*c+ and IDH2R140Q together activate the Hoxa9/Meis1 pathway to drive leukemia in mice [193].

### 3.4. EZH2

Enhancer of zeste homolog 2 (EZH2) incorporates the H3K27me3 mark on its target and causes transcriptional repression [194]. A higher expression level of Ezh2 induces myeloproliferative disease in mice [68]. *Ezh2* null mice show developmental defects and embryonic lethality (Table 1) [69]. Complete ablation of *Ezh2* in the hematopoietic system made the mice susceptible to myelodysplastic disorders [70]. Additional loss of *Tet2* accelerates the disease development in mice [186]. Similarly, *Ezh2* loss cooperates with the *RUNX1*S291fs mutant to accelerate MDS onset in mice [195]. *RUNX1 S291fs/Ezh2* promotes MDS development by activating inflammatory cytokine responses but attenuates leukemia development via PRC1 mediated repression of Hoxa9 [195]. In contrast to the other studies, the loss of *Ezh2* combined with constitutive expression of the NRasG12D mutation-induced leukemic transformation in mice (Table 2; Supplementary Figure S3) [130]. This cooperation amplifies branched-chain amino acid (BCAA) metabolism and enhances mTOR signaling, which is crucial to induce AML in this cooperation model [130]. Deletion of *Ezh2* attenuates the leukemogenicity of KMT2A-MLLT3 expressing cells [196,197]. These data indicate that EZH2 can function either as a tumor suppressor or oncogene, while the determinants of these distinctive roles are not well understood.

### 3.5. ASXL1

The addition of sex combs-like 1 (ASXL1) is an epigenetic modifier that binds to polycomb repressive complex 2 (PRC2) and regulates target gene expression through the H3K27me3 repressive histone modification [198]. Nearly 10% of de novo AML patients show mutations in ASXL1 [199]. ASXL1 mutations also occur in clonal hematopoiesis [200]. 80% of Asxl1^−/−^ mice showed embryonic lethality (Table 1) and the remaining mice displayed features of MDS. Further, heterozygous loss of Asxl1 also induced an MDS-like disease [75]. The retroviral overexpression of C-terminal truncated mutant ASXL1 induced an MDS-like disease in mice (Table 1; Supplementary Figure S1) [71], but knockin Asxl1 mutant mice did not develop any blood disease in mice (Table 1; Supplementary Figure S2) [73]. However, another study showed the development of MDS phenotypes in Asxl1^G643fs/+^ knockin mice (Table 1; Supplementary Figure S2) [74]. Additionally, Asxl1^Y588X^ transgenic mice showed a wide spectrum of myeloid malignancies, including AML, MPNs, and MDS [72]. The discrepancy among ASXL1 mouse models may be due to differences in the expression level of the mutants, different promoters to drive the expression of ASXL1 mutants, and the length/type of ASXL1 mutants. Loss of Asxl1 and NRasG12D collaborates to promote leukemia in mice [201]. Heterozygous loss of both Asxl1 and Nf1 cooperates to accelerate myeloid leukemia in mice (Table 2; Supplementary Figure S3) [131]. The combined loss of both genes induces an MYC-driven transcription signature through H3K4me3 enrichment that prominently contributes to the acceleration of disease development in mice. These reports suggest that the chromatin modifier ASXL1 cooperates with the RAS signaling pathway by NRAS or NF1 mutations to develop leukemia in mice. A recent study also shows that the Asxl1G643W mutant accelerates mutant CEBPA driven AML development in mice (Table 2; Supplementary Figure S3) [136].

### 3.6. ASXL2

Unlike *ASXL1*, *ASXL2* is preferentially mutated in the t(8;21)/RUNX1-RUNX1T1 sub-type of AML patients [202,203]. Half of the *Asxl2*^−/−^ mice show embryonic lethality [76]. *Asxl2* homozygous null mice show myeloid expansion, extramedullary hematopoiesis, and splenomegaly (Table 1) [77]. Li et al. have also shown that homozygous deletion of *Asxl2* causes myeloid skewing to develop MDS-like disease in mice [78]. Further, *Asxl2* loss cooperates with *RUNX1-RUNX1T1* to promote leukemogenesis in mice [204], which supports the clinical occurrence of *ASXL2* mutations in *RUNX1-RUNX1T1* AML patients. Mechanistically, *Asxl2* loss promotes leukemogenesis by increasing chromatin accessibility at the *Hoxa* and *Meis1* loci of *RUNX1-RUNX1T1* transformed cells [204].

## 4. Mouse Models of Nucleophosmin 1 (*NPM1*) in Myeloid Malignancies

Nucleophosmin 1 (*NPM1*) is one of the most frequently mutated genes in AML [2,205] and its mutation is exclusively restricted to myeloid malignancies [206]. However, it is also overexpressed in different solid cancers [206]. NPM1 is a multifunctional protein that primarily resides in the nucleus and plays an active role in different basic biological processes such as ribosome biosynthesis, DNA repair, cellular growth, and stress response [207]. Falini et al. first reported NPM1 mutations in its exon 12 in more than 35% AML cases, characterized by a translocation of the NPM1 protein to the cytoplasm [208]. Insertions or duplications of 4 base pairs in exon 12 disrupt the C terminal nucleolar localization signal (NoLS) that leads to cytoplasmic localization of NPM1, which was later validated in NPM1 transfected cells [209]. NPM1 mutations are always heterozygous [210], which may indicate that the interaction of the wild type and mutant proteins is required for the survival of leukemia cells. While homozygous deletion of *Npm1* shows embryonic lethality in mice (Table 1) [83], heterozygous loss of *Npm1* mostly predisposes to MDS in mice [83,84]. This implies that wild-type NPM1 is required for cell survival and supports the biological importance of the heterozygous mutation status in AML patients. Different models of NPM1 mutations show that it induces myeloproliferative disorders. For example, expression of the cytoplasmic *NPM1* mutant (*NPM*c+) under the human myeloid-specific MRP8 promotor or in a conventional knockin mouse model induced myeloproliferative disease [79,80]. Vassiliouet al. and Mallardo et al. have observed a late AML onset in some NPM1 mutated knockin mice (Table 1; Supplementary Figure S3) [81,82]. Two different cooperation models of mutant *NPM1* with two different signal transduction genes, FLT3-ITD [82,121,122] and NRASG12D [129], showed rapid leukemia onset in mice (Table 2; Supplementary Figure S3). In the double mutant mouse models, Dovey et al. have shown that the Npmc+*/Flt3*-ITD combination leads to AML with a shorter latency in mice compared to the Npmc+/NrasG12D combination [129]. In these cooperation models, a dependence was noted on the Hoxa network for the maintenance of leukemic cells [129], and these results coincide with upregulation of the HOXA genes in NPM1 mutant AML patients [211]. The other common FLT3 mutation, FLT3-TKD, also synergizes with mutated NPM1 and induces a short-latency AML in mice [21]. Clinically, NPM1 mutations also co-occur with both FLT3-ITD and FLT3-TKD mutations. Another study has discovered cooperation of NPM1 and IDH2 mutations for leukemia development in mice through activation of the Hoxa9/Meis1 pathway, where NPMc and IDH2/R140Q increase the expression of Hoxa9 and Meis1, respectively [193]. Chou et al. have observed that the NPM1 mutation downregulates CXCR4/CXCL12 pathway genes to induce myeloproliferation in mice and a similar observation has been made in human *NPM1* positive AML patients [80]. Another study has shown that the *NPM1* mutation drives *Dnmt3a* mutant clonal hematopoiesis to AML in mice [134]. In this line, a recent interesting study has demonstrated that disruption of the KMT2A-Menin chromatin complex using a small molecule inhibitor (VTP-50469) significantly increases the leukemic latency of *Npm1*c+/*Dnmt3a* double mutant mice [212]. Additionally, combined menin and FLT3 inhibition show potent antileukemic effect in NPM1 and FLT3 double mutant primary AML patient cells [213].

## 5. Mouse Models of Transcription Factor Genes in Myeloid Malignancies

A transcription factor regulates the expression of its target gene by controlling its transcription. Myeloid transcription factors are usually differentially expressed between healthy and disease states. Mutations in different transcription factors have been discovered in AML patients.

### 5.1. CEBPA

The CCAAT enhancer-binding protein alpha (*CEBPA*) gene encodes two isoforms: the 42 kDa isoform (p42) and the 30 kDa isoform (p30) [214]. Approximately one-tenth of AML patients display *CEBPA* mutations [215,216]. The most predominantly occurring mutations involve loss of p42 [217]. Homozygous *Cebpa* knockout mice die shortly after birth (Table 1) [86]. Conditional knockout of *Cebpa* in adult mice shows a selective block of granulocytic development and accumulation of blasts in the bone marrow [218]. Interestingly, mice lacking the p42 isoform but retaining the p30 isoform develop AML. Biallelic loss of the p42 isoform rapidly induced AML, suggesting a tumor suppressor function of the p42 isoform [87]. This result explains why mutations in AML patients frequently occur in the p42 isoform. *CEBPA* mutations broadly occur as two types of mutations: N terminal mutations and C terminal mutations. N terminal mutations lead to loss of the p42 isoform and C terminal mutations are located in the basic region-leucine zipper DNA binding domain. AML patients with biallelic CEBPA mutations show an N terminal mutation on one allele and a C terminal mutation on the other allele. In this line, Bereshchenko et al. have shown that mice carrying both N terminal and C terminal CEBPA mutations show rapid induction of AML (Table 1; Supplementary Figure S2) [85]. An additional ASXL1-G643W mutation accelerated the development of AML in vivo [136]. Further, mutations in CEBPA and granulocyte colony-stimulating factor receptor (CSF3R) show synergy in the development of AML in mice, where CSF3R signaling induced both proliferation and differentiation, and the Cebpa mutation blocked differentiation through inactivation of differentiation-associated enhancers [219]. Another mouse study showed that wild-type CEBPA is required for KMT2A-MLLT1 driven leukemia through activation of Hoxa9/Meis1 [220]. In this line, Collins et al. have shown that inactivation of Cebpa impairs Hoxa9/Meis1-mediated leukemogenesis [221]. These studies prove that wildtype CEBPA is indispensable for Hoxa9/Meis1 mediated transformation and provides an explanation for the absence of *CEBPA* null mutations in AML patients.

### 5.2. RUNX1

RUNX1 (AML1) belongs to the Runt-related transcription factor (RUNX) family of proteins that recognize 5′-TGTGGT-3′ and binds to this motif by forming a complex with core-binding factor beta (CBFβ) [222]. RUNX1 controls hematopoiesis by regulating the transformation of hematopoietic stem cells to differentiated cells [223]. *Runx1* is required for fetal hematopoiesis, and its loss causes embryonic lethality in mice (Table 1) [89]. In contrast, *Runx1* is not essential for adult hematopoiesis and a conditional *Runx1* knockout in adult mice showed a myeloproliferative phenotype [224]. Genetic or chromosomal alterations of *RUNX1* frequently occur in AML patients [222,225]. Retroviral overexpression of two mutants of RUNX1, D171N, and S291fsX300, induce an MDS phenotype in mice (Table 1; Supplementary Figure S1) [88,226]. Further, the RUNX1-D171N mutation collaborates with overexpression of Evi1 to induce leukemia in mice [88]. Behrens et al. demonstrated that FLT3-ITD collaborates with RUNX1 mutations to induce an aggressive AML in mice (Table 2; Supplementary Figure S3) [20]. Cooperation of Runx1 deficiency and U2af1S34F mutation induces AML in mice [111]. RUNX1 also cooperates with ASXL1 to accelerate leukemogenesis through activating the HIF1-α pathway [227]. Runx1 has also been shown to be required for *KMT2A-MLLT3* leukemogenesis in mice [228].

### 5.3. MYC

The MYC gene family consists of *C-MYC*, *N-MYC*, and *L-MYC* and encodes proteins that function as transcription factors [229]. MYC proteins are tightly regulated in the healthy state but are dysregulated in cancers [230]. Dysregulated MYC expression, rearrangements (particularly in lymphoma), and overexpression have been identified in hematological neoplasms [231]. Germline ablation of murine *C-Myc* causes embryonic lethality (Table 1) [92]. Overexpression mouse models of *C-Myc*, and *N-Myc* show rapid AML development (Table 1; Supplementary Figure S1) [90,91], underscoring the importance of *MYC* overexpression in AML patients.

### 5.4. BCOR

*The BCOR* gene, which encodes the BCL-6 corepressor (BCOR), is located on the X chromosome. It acts as a corepressor for BCL6 to cause BCL6 mediated transcriptional repression [232]. Approximately 4% of CN-AML patients show mutations in the BCOR gene [233]. *Bcor* knockout male mice die before birth (Table 1) [95]. (*Bcor^ΔE4/y^*) mice that lack the BCL6 binding domain develop T-ALL [93]. Moreover, after the deletion of exons 9 and 10, BCOR fails to interact with polycomb repressive complex 1.1 and also causes lethal T-ALL in mice. However, *Bcor^ΔE9−10/y^* shows a proliferative advantage in the myeloid compartment and combined with loss of *Tet2*, these mice develop MDS [94]. Concurrent knockout of *Bcor* and *Dnamt3a* cause acute erythroid leukemia in mice (Table 2; Supplementary Figure S3) [135]. The combined loss of *Bcor* and *Dnamt3a* shows expression changes in Gata genes and p53 family members that may contribute to their collaboration [135]. *Bcor* loss and KrasG12D cooperate to induce AML in mice (Table 2; Supplementary Figure S3), and Hoxa9 is required for *Bcor^ΔE9−10^Kras^G12D^* tumors [126]. MLLT3, a fusion partner of KMT2A, directly interacts with BCOR, and loss of this interaction abrogates the leukemogenic potential of *KMT2A-MLLT3/MLL-AF9* in mice [234]. Loss of this interaction results in the reduction of c-Myc expression.

### 5.5. CUX1

The CUT-like homeobox 1 (*CUX1)* gene, a homeodomain-containing transcription factor, is present on chromosome 7, and mutations are frequently reported in del(7q) AML patients [235,236]. *CUX1* mutations are often haploinsufficient in del(7q) AML patients, suggesting a role as a tumor suppressor. Homozygous *Cux1* null mice show a high postnatal death rate (Table 1) [97]. shRNA-mediated knockdown of *Cux1* induces MDS in mice. Additionally, different expression levels of Cux1 show different disease phenotypes, suggesting that the development of the disease depends on the Cux1 dose [237]. Transgenic mice expressing the *p75 Cux* isoform, which is overexpressed in breast cancers, develop a myeloproliferative disease–like myeloid leukemia (Table 1; Supplementary Figure S1) [96]. *Cux1* haploinsufficiency combined with the *Flt3-ITD* mutation induces AML and CMML in mice (Table 2; Supplementary Figure S3) [30]. These mice show apoptosis defects in the hematopoietic stem and progenitor cell compartments. Cux1 inactivation increases the expression of CASP8 and FADD-like apoptosis regulator (CFLAR), which may contribute to the defect in apoptosis.

### 5.6. SETBP1

*SETBP1* mutations are frequently found in different myeloid malignancies such as MDS, JMML, and AML [238,239]. Although *SETBP1* mutations are less frequent in primary AML patients, more than 15% of secondary AML patients display mutations in *SETBP1* [240]. Setbp1 overexpression induces myeloid leukemia in mice by transcriptional repression of Runx1 (Table 1; Supplementary Figure S1) [98]. However, overexpression of human SETBP1-D868N only causes splenomegaly (Table 1; Supplementary Figure S1) [99]. Transplantation of hematopoietic cells expressing both SETBP1 and ASXL1 mutants causes AML in mice (Table 2; Supplementary Figure S3) [99]. This study showed that the addition of mutated SETBP1 further enhances Hoxa9 and Hoxa10 expression in ASXL1 mutant cells [99]. Using the *Sleeping Beauty* transposon system, Pacharne et al. have shown that all *FLT3-ITD* mice developed AML with *Setbp1* being the most frequent integration site. (Table 2; Supplementary Figure S3) [29]. It was demonstrated that Setbp1 overexpression activates the *HOXA* gene signature and *Flt3*^ITD/+^*/Setbp1*^IM+^ AML is vulnerable to Kdm1a and Brd3 inhibition [29]. These studies indicate that Setbp1 contributes to leukemogenesis predominantly through the regulation of Hox genes.

### 5.7. PHF6

Mutations in the Plant homeodomain finger gene 6 (*PHF6*) gene are commonly observed in Börjeson-Forssman-Lehmann syndrome patients [241]. *PHF6* mutations are more frequent in T-ALL (about 20%) patients but less frequent in adult AML (about 3%) patients [242]. *PHF6* mutations are associated with reduced overall survival in AML [243]. Germline *Phf6* deletion causes perinatal death in male mice (Table 1) [101]. Conditional knockout of *Phf6* in hematopoietic cells causes myelodysplasia-like disease in mice, suggesting its role as a tumor suppressor in leukemia pathogenesis [100]. PHF6 mutations frequently co-occur with RUNX1 mutations in AML [171,244], but no synergistic model of both mutations is described yet.

## 6. Mouse Models of Tumor Suppressor Genes in Myeloid Malignancies

Like in other cancers, genetic abnormalities in tumor suppressor genes were also reported in AML patients. *WT1* and *TP53* mutations are frequently recurring tumor suppressor mutations in AML patients.

### 6.1. WT1

Wilms tumor 1 (*WT1*) was initially discovered as a tumor suppressor gene, but later it was also identified as an oncogene in various cancers. Although *WT1* is dispensable for fetal hematopoiesis [245], it plays a vital role in adult hematopoiesis [246]. Approximately 6–15% of AML patients show mutations in the *WT1* gene [247,248,249,250]. Mutations of this gene result in loss of function of the corresponding protein [248,251], which contributes to oncogenic transformation. Complete loss of *Wt1* causes embryonic lethality in mice [102]. Heterozygous Wt1R394W knockin mice display an MDS phenotype (Table 1; Supplementary Figure S2), but Flt3^+/ITD^/Wt1^+/R394W^ mice show a more aggressive phenotype with few AML phenotypes [26]. This suggests that a third hit is required for fully penetrant AML development in mice. In another study using a conditional knockout model, Pronier et al. have shown that *Wt1* haploinsufficient mice develop T-ALL, and *Wt1* haploinsufficient mice carrying an additional *FLT3-ITD* mutation develop a lethal AML (Table 2; Supplementary Figure S3) [27]. WT1 acts as a cofactor for TET2 in mediating 5-hydroxymethylation of cytosines (5-hmC), therefore deletion of *WT1* or a mutation in *WT1* disrupts this pathway [252,253]. Mutations in either of these genes disrupt the formation of 5-hydroxymethylation of cytosines (5-hmC), which is supported by the exclusive occurrence of *WT1* and *TET2* mutations in AML patients [243].

### 6.2. TP53

*TP53* is a tumor suppressor gene that is frequently dysregulated in various cancers. Approximately 8 percent of AML patients have been reported to be diagnosed with a *TP53* mutation [254]. Although studies have shown that the frequency of *TP53* mutations is lowest in AML compared to other cancers, AML patients carrying *TP53* mutations show shorter survival compared to *TP53* wildtype patients in the TCGA data set [2,254,255], underscoring the importance of *TP53* in AML pathogenesis and prognosis. TP53 is highly expressed in HSCs and mice lacking *Trp53*, the equivalent of *TP53*, show an increase in the HSC population [256,257,258]. Mice carrying *TP53* mutations (p53R172H, p53R172P, p53R270H) or a full deletion are often prone to hematopoietic neoplasms such as lymphoma, leukemia, and T cell or B cell malignancies (Table 1; Supplementary Figure S1) [103,105,106,259,260,261,262]. Hanel et al. have shown the early onset of hematologic disease in R248Q knockin mice compared to G245S mutated mice and null mice (Table 1; Supplementary Figure S2) [104], and its inactivation exerts chemoresistance in mice [155], indicating different oncogenic strengths among *TP53* mutations. Several oncogenic cooperation mouse models show that mutations in *TP53* or knockout of *TP53* aggravate AML. Basova et al. have shown that deletion of *P53* accelerates AML that is induced by the mutant transcription factor PU. 1 [263]. Additional models show that *P53* inactivation cooperates with NRASG12D or KrasG12D to drive an aggressive AML phenotype in mice (Table 2; Supplementary Figure S3) [124,127]. These mouse model results are consistent with the clinical co-occurrence of *P53* mutations with an aberrant RAS signaling pathway. It suggests that the restoration of the tumor suppressor protein function can be an effective strategy to treat AML.

## 7. Mouse Models of Spliceosome Complex Genes in Myeloid Malignancies

Splicing removes non-coding introns from precursor messenger RNA which is mediated by the spliceosome complex. Mutations in these genes cause aberrant splicing [264]. Splicing factor mutations more often appear in the founding clone rather than a subclone in the evolution of MDS [265,266] and are also detected in aging individuals lacking any hematological malignancy [267].

### 7.1. SRSF2

The most frequent splicing factor mutated in AML is SRSF2, which prominently occurs in the Proline 95 residue [268]. Homozygous knockout of *Srsf2* causes embryonic lethality in mice [107]. Mice transplanted with murine bone marrow cells either retrovirally overexpressing the wildtype SRSF2 or the mutant SRSF2P95H variant do not develop any myeloid malignancy [107]. However, Srsf2P95H/wt knockin mice develop MDS (Table 1; Supplementary Figure S2) [108], while only a mild phenotype was observed in another knockin model [109]. A mechanistic study by Kim et al. shows that the mutant SRSF2 exhibits altered RNA binding activity that leads to the degradation of EZH2 by nonsense-mediated decay [108]. In another knockin mouse model, SRSF2P95H mutant cells were found to rely on the wild-type allele for their survival [269]. The SRSF2 mutation cooperates with the IDH2 mutation and causes a lethal MDS in mice. It causes aberrant splicing of INTS3 (Integrator Complex Subunit 3) that resulted in its reduced expression through nonsense-mediated decay [270]. The collaboration of loss of Runx1 and the Srsf2P95H mutation causes MDS in mice [271].

### 7.2. U2AF1

U2AF1 recognizes and interacts with the AG nucleotides at the 3’ splice site and the common U2AF1S34 mutation alters this interaction [272]. Biallelic deletion of *U2af1* shows embryonic lethality in mice [112]. A doxycycline-inducible transgenic mouse model of mutant U2AF1 (S34F) did not develop any dysplasia, MDS, or AML, while leukopenia and progenitor cell expansion were the only phenotypes [110]. However, *U2AF1*(S34F) knockin mice showed MDS features such as cytopenia and dysplasia (Table 1; Supplementary Figure S2) [111]. The *Runx1* deletion and the *U2af1*(S34F) oncogenic mutation cooperate to induce AML in mice (Table 2; Supplementary Figure S3) [111]. A recent interesting study demonstrated that mutant U2AF1 leukemia cells depend on the wildtype U2AF1 allele for their survival [112], explaining why splice factor mutations are always found in the heterozygous condition in patients.

### 7.3. SF3B1

While complete ablation of *Sf3b1* leads to embryonic lethality in mice (Table 1) [116], haploinsufficiency caused MDS in mice [115]. This shows that the presence of the wild-type allele is important for its mutant counterpart. Moreover, hematopoietic specific SF3B1K700E expression showed some MDS-like features in two different knockin mouse models (Table 1; Supplementary Figure S2) [113,114]. Additionally, *Sf3b1*^+/K700E^ *Tet2*^−/−^ cooperation mouse model showed early onset of MDS [114].

The currently available mouse models of splicing factor genes show that the heterozygous state of the splicing factor mutations is important for the induction of myeloid malignancies. Currently, no mouse model shows leukemia induction by a splice factor mutation and there are very limited collaboration studies to show the synergy between splicing factor genes and other frequently mutated genes in AML. The dependency of spliceosome mutations on the wildtype allele suggests that the synthetic lethality properties of spliceosome mutations can be exploited in the future for drug development.

## 8. Mouse Models of Cohesin Complex Genes in Myeloid Malignancies

The cohesin complex is a ring-like structure that is formed by SMC1A, SMC3, RAD21, and STAG1/2 [273]. This cohesin complex holds the sister chromatids together and thus plays an important role in chromosome segregation during cell division [274]. Therefore, cohesin mutations may lead to aneuploidy. Additionally, the cohesin complex is involved in modulating gene expression through genome organization by increasing DNA accessibility for transcription factors [275,276]. However, mutations in the cohesin complex do not cause aneuploidy in AML, suggesting that the control of the gene expression function by the cohesin complex is crucial for leukemogenesis [277]. The frequency of mutations in members of the cohesin complex is around 10% in AML patients [274,278]. Cohesin gene mutations are rarely seen as solitary mutations in AML and often co-occur with other mutations such as *NPM1*, *DNMT3A*, *TET2*, or *RUNX1* [278,279,280]. The mechanistic role of these mutations in leukemic transformation in mouse models is largely unexplored. Biallelic deletion of cohesin genes *Stag1*, *Stag2*, *Smc3*, and *Rad21* in mice is reported to be embryonically lethal, indicating its importance in the normal embryonic development process [117,118,119,120]. Further, abrogating the function of the cohesin complex in mice using shRNA-mediated knockdown shows features of myeloproliferative neoplasms [281]. Mutations in cohesin complex genes are usually mutually exclusive, implicating that a mutation in any of the members of the cohesin complex is sufficient to disrupt the function of the whole cohesin complex in leukemia [274,278]. Unlike *Stag1* deficient hematopoietic cells, *Stag2* deficient hematopoietic cells show an increase in self-renewal activity. Complete ablation of *Stag2* in mice induces myeloid dysplasia and reduced differentiation to the B cell lineage through transcriptional control of the lineage-specific genes *Ebf1* and *Pax5* [282]. The combined loss of both *Stag1* and *Stag2* causes pancytopenia and bone marrow aplasia in mice [282]. Additionally, the combined knockout of *Stag2* and *Runx1* recapitulates an MDS phenotype in mice [283]. This study also indicates that codeletions of *STAG2/RUNX1* disrupt chromatin looping, which regulates super enhancer-associated genes such as *Hoxa9* and basal pausing genes involved in IFN and inflammatory responses that may contribute to leukemogenesis [283]. This model also explains the frequent co-occurrence of *STAG2* and *RUNX1* mutations in AML [284]. Homozygous loss of Smc3 induces bone marrow aplasia, but the haploinsufficiency of Smc3 shows the renewal of stem cell activity. Further, the combination of *Smc3^−/+^* and FLT3-ITD induces AML in mice with enrichment of the *STAT5A* gene signature (Table 2; Supplementary Figure S3) [19]. This suggests that reducing the level of Smc3a provides an accessible chromatin structure for Stat5-mediated transcription [19]. In summary, cohesin gene mutations are loss of function mutations, which are crucial for leukemogenesis.

## 9. Conclusions

AML shows a diverse genomic landscape and patients without driver mutations display low blast counts and better outcomes, indicating the importance of genomic lesions in AML pathogenesis [2]. Mouse models are considered to be ideal tools to study the in vivo pathogenesis and to understand the underlying biological interactions among driver mutations. Here we summarized the available evidence of the biological function of mutated genes available from mouse models. Frequent co-occurrence and exclusiveness of mutations in AML patients highlight the importance of gene-gene interactions in AML development. In Supplementary Figure S3, we show a map of genes that functionally cooperate in vivo. This illustrates that mutations in signaling genes are the most frequent cooperation partners of mutations in the other functional pathways. It also shows that signaling genes almost always work by overexpression/knockin (red or grey color in Supplementary Figure S3), while the cooperation partners in the other pathways are either activated (red) or inactivated (grey). This provides the basis to identify functional dependencies that may lead to novel therapeutic approaches. In addition, Supplementary Figure S3 shows the data gaps that should be closed in the near future.

Hotspot mutations in the same gene can differ in their oncogenic potential and preferential cooperation partners. We discuss how different oncogenic mutations in the same gene have a variable leukemogenic potential. ITD or TKD mutations in the *FLT3* gene show the potential to induce different hematologic malignancies. Similarly, we discuss differences in oncogenic potential for hotspot mutations in *TP53* and *PTPN11*. In a large patient cohort, it is reported that *NPM1* mutations preferentially co-occur with NRASG12/13 but not with NRASQ61. Hot spot mutations in *IDH2* and *FLT3* also show different preferential partners [2]. This indicates that functional consequences of mutations in the same gene may have a different biologic consequence in the development of AML and therefore show distinct co-mutation patterns. AML patients are usually identified with more than one genomic abnormality. We describe how the cooperative effect of two different mutations can lead to an aggressive disease in vivo. We also highlight how a novel pathway emerges as a synergistic effect of two different mutations. TP53 lesions with complex karyotype AML constitute an adverse risk group in the AML cohort. These patients often lack RAS pathway mutations, indicating redundancy in RAS mutations and loss of RAS regulators by chromosomal alterations [2]. AML patients with ASXL1 and SRSF2 mutations show a very poor prognosis [2]. However, the additive biological effect of these two mutations has not been clearly demonstrated in mouse models and is not clearly understood yet.

In Figure 1 we show the functional synergy of mutations in different pathways. Synergy is rarely seen among genes of the same functional group. Cell signaling genes are the most promiscuous gene class that collaborates with mutations in 5 of the 6 other gene classes. Mutated genes in epigenetic modifiers and transcription factors collaborate with mutations in 3 other gene classes. Mutated *NPM1* collaborates with mutations in 2 other gene classes and mutations in the cohesin complex, spliceosome complex, and tumor suppressor genes functionally synergize with only one other gene class. This suggests that mutations in signaling genes are most variable and least specific regarding their collaboration partners and may explain why they often occur as subclonal mutations in AML.

Identifying recurring mutations with modern sequencing technologies and studying the pathophysiological effect of those mutations using mouse models will provide more insights into the biology of AML that will eventually lead to the discovery of more effective treatments for AML patients.

## Figures and Tables

**Table 1:** Overexpression, knockin and knockout effect of frequently mutated genes in AML.

Group	Genes	Overexpression/ Transgenic Mice Model	Knockin	Knockout
Cell signaling genes	FLT3	FLT3 WT-ND FLT3 ITD -MPN [13] FLT3-ITD (Vav promoter) -MPN and B- or T-lymphoid disorders [15] FLT3 TKD -Lymphoid disorder [14]	FLT3^wt/ITD^-MPN [17] FLT3/D835Y- MPN, lymphomas and sarcomas [18] Flt3^+/ITD^ and Flt3^ITD/ITD^ -CMML [16]	FLT3^−/−^ -Viable but deficiencies in B lymphoid progenitor [12]
	KIT	hKIT wt -ND hKIT D816V -ND hybrid C-KIT D816V-MPN [35] HyC-KIT N822K-MPN [36]	NA	c-Kit^−/−^ -Postnatal death [37]
	KRAS	KRAS G12D-ND [38]	KRAS G12D- MPN [39,40]	Kras^−/−^ -Embryonic lethal [41,42]
	NRAS	NRAS G12D (MSCV promoter) -ND [38,43] NRASD12 -CMML and AML [44] NRAS G12D (hMRP8 promoter) -Hyperkeratotic skin lesions [45] Mo-MuLV Nras G12D -MPNs [46]	Nras G12D -MPN [47]	Nras^−/−^ -Viable and no defect in hematopoiesis [48]
	NF1	NA	NA	Nf1^−/−^ -Embryonic lethality [49] Nf1^+/−^ -Various tumors [50]
	PTPN11	PTPN11 wt -ND PTPN11 E76K -JMML PTPN11 D61Y -JMML [51]	Ptpn11 D61Y [52] -MPN Ptpn11 E76K/+ -MPN [53]	Ptpn11^−/−^ -Embryonic lethal [54]
Epigenetic modifiers	DNMT3A	DNMT3A Wt-ND DNMT3A R882H -CMML [55]	Dnmt3a R878H/WT -AML [56]	Dnmt3a^−/−^ -Viable [57] Conditional Dnmt3a^−/−^ -MDS/ MPN [58]
	TET2	NA	NA	TET2^−/−^ -wide spectrum myeloid malignancies [59,60] Conditional Tet2^+/−^ -EMH [61]
	IDH1	IDH1 WT-ND IDH1 R132C -ND [62]	IDH1 R132H -EMH and Splenomegaly [63]	Idh1^−/−^ -Viable [64]
	IDH2	IDH2 R140Q -EMH, Splenomegaly [24]	Idh2 R140Q -ND [65,66]	Idh2^−/−^-Viable [67]
	EZH2	EZH2 wt -MPN [68]	NA	Ezh2^−/−^ -Embryonic lethal [69] Hematopoietic Ezh2^−/−^ -MDS [70]
	ASXL1	C -terminal truncated mutant ASXL1 -MDS [71] Asxl1 Y588X -AML/MDS/MPN [72]	Asxl1 G643fs -ND [73] Asxl1 G643fs/+ -MDS [74]	Asxl1^−/−^ -Embryonic lethal/MDS Asxl1^+/−^ -MDS [75]
	ASXL2	NA	NA	Asxl2^−/−^ -Partial Embryonic lethal/Mild BM disorders/MDS [76,77,78]
Nucleophosmin 1	NPM1	NPM1c+ -myeloproliferation [79] NPM1 -ND [79]	Npm1 wt/c+ -MPN some mice [80] late AML onset in some mice [81,82]	Npm1^+/−^ -MDS [83,84] Npm1^−/−^ -Embryonic lethal [83]
Transcription factors	CEBPA	NA	CEEBPA K313KK/Lp30 Retxn -AML [85]	CEBPA^−/−^ -Postnatal death [86] CEBPA p42^+/−^ -ND CEBPA p42^−/−^ -AML [87]
	RUNX1	RUNX1 D171N and S291fsX300 -MDS [88]	NA	Runx1^−/−^ -Embryonic lethal [89] with hematopoietic defect
	MYC	C-Myc -AML [90] N-Myc -AML [91]	NA	C-Myc^−/−^ Embryonic lethal [92]
	BCOR	NA	Bcor ΔE4/y -TALL [93] Bcor ΔE9−10/y -TALL [94]	Bcor^−^*^/Y^* -Male embryonic lethality [95]
	CUX1	p75 CUX -MPN [96]	NA	Cux1^−/−^ -Postnatal death [97]
	SETBP1	Setbp1 -Myeloid leukemia [98] SETBP1-D868N Splenomegaly [99]	NA	NA
	PHF6	NA	NA	Conditional hematopoietic knockout -Myelodysplasia-like disease [100] Phf6^−/Y^ -Perinatal lethality in males [101]
Tumor suppresors	WT1	NA	Wt1+/R394W -MDS [26]	Wt1^−/−^ -Embryonic lethal [102] Wt1^fl/+^ -T-ALL [27]
	TP53	NA	p53 R172H -Lymphoma, leukemia or mix [103] p53 R248Q -T cell/B cell lymphoma, solid tumors [104]	P53^−/−^ -Majorly lymphoma [105,106]
Spliceosome complex	SRSF2	SRSF2 WT -ND SRSF2 P95H -ND [107]	Srsf2 P95H/wt -Myelodysplasia [108] impaired hematopoietic stem cell functions [109]	Srsf2^−/−^ -Embryonic lethal [107]
	U2AF1	U2AF1 S34F -Leukopenia [110]	U2af1 S34F/WT -MDS-like phenotype [111]	U2af1^−/−^ -Embryonic lethal [112]
	SF3B1	NA	Sf3b1 K700E/+ -Anemia [113] Sf3b1+/K700E -MDS [114]	Sf3b1^+/−^ -MDS [115] Sf3b1^−/−^ -Embryonic lethal [116]
Cohesin complex	RAD21	NA	NA	Rad21^−/−^ -Embryonic lethal [117]
	STAG1	NA	NA	Stag1^−/−^ -Embryonic lethal [118]
	STAG2	NA	NA	Stag2^−/−^ -Embryonic lethal [119]
	SMC3	NA	NA	Smc3^−/−^ -Embryonic lethal [120]

ND-no disease; EMH-extramedullary hematopoiesis; MPD-myeloproliferative disease; MPN-myeloproliferative neoplasm; MDS-myelodysplastic syndromes; JMML-juvenile myelomonocytic leukemia; CMML-chronic myelomonocytic leukemia; NA-not available.

**Table 2:** Synergistic AML models of frequently mutated genes in AML.

Group	Genes	Synergistic Genes in the Development of AML in Mice
Cell signaling genes	FLT3	SMC3−/+ [19] RUNX1 [20] NPM1c+ [82,121,122] Dnamt3a−/− [22,23] IDH2 R140Q [24] or IDH2 R172K [25] Wt1fl/+ [26,27] TET2−/− [28] Cux1+/− [30] Setbp1 [29]
	KIT	NA
	KRAS	Dnmt3a−/− [123] P53 [124] Nf1 [125] Bcor ΔE9−10 [126]
	NRAS	P53−/− [127] Dnmt3a R878H [128] IDH2 R140Q or IDH2 R172K [25] Npm1cA [129] EZH2−/− [130]
	NF1	Asxl1+/− [131] Kras G12D [125]
	PTPN11	NA
Epigenetic modifiers	DNMT3A	Kras G12D/+ [123] FLT3 ITD [22,23] Nras G12D [128,132] IDH2 neomorphic [133] Npm1cA [134] Bcor−/− [135]
	TET2	FLT3 ITD [28]
	IDH1	NA
	IDH2	Nras G12D [25] FLT3 ITD [24,25] Dnmt3a−/− [133]
	EZH2	NRAS G12D [130]
	ASXL1	CEBPA [136] Cebpa D/p30 [136] SETBP1 D868N [99] Nf1^+/–^ [131]
	ASXL2	NA
Nucleophosmin 1	NPM1	FLT3 ITD [82,121,122] FLT3 TKD [21] NRAS G12D [129] Dnmt3a R878H [134]
Transcription factors	CEBPA	Asxl1 G643W [136]
	RUNX1	FLT3 ITD [20] U2af1 S34F [111]
	MYC	NA
	BCOR	Dnamt3a−/− [135] Kras G12D [126]
	CUX1	Flt3 ITD [30]
	SETBP1	ASXL1 MT [99] FLT3 ITD [29]
	PHF6	NA
Tumor suppressors	WT1	FLT3 ITD [26,27]
	TP53	NARS G12D [127] Kras G12D [124]
Spliceosome complex	SRSF2	NA
	U2AF1	Runx1^F/F^ [111]
	SF3B1	NA
Cohesin complex	RAD21	NA
	STAG1	NA
	STAG2	NA
	SMC3	FLT3 ITD [19]

## Supplementary Materials

The following are available online at https://www.mdpi.com/article/10.3390/cancers13246192/s1. Figure S1. Disease induction by frequently mutated genes in transgenic/overexpression mouse models. Figure S2. Disease induction by frequently mutated genes in knockin mouse models. Figure S3. Map of genes that functionally cooperate in vivo.

## References

[1] Saultz J.N., Garzon R. (2016). Acute Myeloid Leukemia: A Concise Review. J. Clin. Med..

[2] Papaemmanuil E., Gerstung M., Bullinger L., Gaidzik V.I., Paschka P., Roberts N.D., Potter N.E., Heuser M., Thol F., Bolli N. (2016). Genomic Classification and Prognosis in Acute Myeloid Leukemia. N. Engl. J. Med..

[3] Kantarjian H., Kadia T., DiNardo C., Daver N., Borthakur G., Jabbour E., Garcia-Manero G., Konopleva M., Ravandi F. (2021). Acute myeloid leukemia: Current progress and future directions. Blood Cancer J..

[4] Ding L., Ley T.J., Larson D.E., Miller C.A., Koboldt D.C., Welch J.S., Ritchey J.K., Young M.A., Lamprecht T., McLellan M.D. (2012). Clonal evolution in relapsed acute myeloid leukaemia revealed by whole-genome sequencing. Nature.

[5] Döhner H., Weisdorf D.J., Bloomfield C.D. (2015). Acute Myeloid Leukemia. N. Engl. J. Med..

[6] Voso M.T., Ottone T., Lavorgna S., Venditti A., Maurillo L., Lo-Coco F., Buccisano F. (2019). MRD in AML: The Role of New Techniques. Front. Oncol..

[7] Carbonell D., Suárez-González J., Chicano M., Andrés-Zayas C., Triviño J.C., Rodríguez-Macías G., Bastos-Oreiro M., Font P., Ballesteros M., Muñiz P. (2019). Next-Generation Sequencing Improves Diagnosis, Prognosis and Clinical Management of Myeloid Neoplasms. Cancers.

[8] Almosailleakh M., Schwaller J. (2019). Murine Models of Acute Myeloid Leukaemia. Int. J. Mol. Sci..

[9] Small D. (2006). FLT3 mutations: Biology and treatment. Hematol. Am. Soc. Hematol. Educ. Program..

[10] Daver N., Schlenk R.F., Russell N.H., Levis M.J. (2019). Targeting FLT3 mutations in AML: Review of current knowledge and evidence. Leukemia.

[11] Kennedy V.E., Smith C.C. (2020). FLT3 Mutations in Acute Myeloid Leukemia: Key Concepts and Emerging Controversies. Front. Oncol..

[12] Mackarehtschian K., Hardin J.D., Moore K.A., Boast S., Goff S.P., Lemischka I.R. (1995). Targeted disruption of the flk2/flt3 gene leads to deficiencies in primitive hematopoietic progenitors. Immunity.

[13] Kelly L.M., Liu Q., Kutok J.L., Williams I.R., Boulton C.L., Gilliland D.G. (2002). FLT3 internal tandem duplication mutations associated with human acute myeloid leukemias induce myeloproliferative disease in a murine bone marrow transplant model. Blood.

[14] Grundler R., Miething C., Thiede C., Peschel C., Duyster J. (2005). FLT3-ITD and tyrosine kinase domain mutants induce 2 distinct phenotypes in a murine bone marrow transplantation model. Blood.

[15] Lee B.H., Williams I.R., Anastasiadou E., Boulton C.L., Joseph S.W., Amaral S.M., Curley D.P., Duclos N., Huntly B.J., Fabbro D. (2005). FLT3 internal tandem duplication mutations induce myeloproliferative or lymphoid disease in a transgenic mouse model. Oncogene.

[16] Lee B.H., Tothova Z., Levine R.L., Anderson K., Buza-Vidas N., Cullen D.E., McDowell E.P., Adelsperger J., Fröhling S., Huntly B.J. (2007). FLT3 mutations confer enhanced proliferation and survival properties to multipotent progenitors in a murine model of chronic myelomonocytic leukemia. Cancer Cell.

[17] Li L., Piloto O., Nguyen H.B., Greenberg K., Takamiya K., Racke F., Huso D., Small D. (2008). Knock-in of an internal tandem duplication mutation into murine FLT3 confers myeloproliferative disease in a mouse model. Blood.

[18] Bailey E., Li L., Duffield A.S., Ma H.S., Huso D.L., Small D. (2013). FLT3/D835Y mutation knock-in mice display less aggressive disease compared with FLT3/internal tandem duplication (ITD) mice. Proc. Natl. Acad. Sci. USA.

[19] Viny A.D., Ott C.J., Spitzer B., Rivas M., Meydan C., Papalexi E., Yelin D., Shank K., Reyes J., Chiu A. (2015). Dose-dependent role of the cohesin complex in normal and malignant hematopoiesis. J. Exp. Med..

[20] Behrens K., Maul K., Tekin N., Kriebitzsch N., Indenbirken D., Prassolov V., Muller U., Serve H., Cammenga J., Stocking C. (2017). RUNX1 cooperates with FLT3-ITD to induce leukemia. J. Exp. Med..

[21] Rudorf A., Muller T.A., Klingeberg C., Kreutmair S., Poggio T., Gorantla S.P., Ruckert T., Schmitt-Graeff A., Gengenbacher A., Paschka P. (2019). NPM1c alters FLT3-D835Y localization and signaling in acute myeloid leukemia. Blood.

[22] Meyer S.E., Qin T., Muench D.E., Masuda K., Venkatasubramanian M., Orr E., Suarez L., Gore S.D., Delwel R., Paietta E. (2016). DNMT3A Haploinsufficiency Transforms FLT3ITD Myeloproliferative Disease into a Rapid, Spontaneous, and Fully Penetrant Acute Myeloid Leukemia. Cancer Discov..

[23] Poitras J.L., Heiser D., Li L., Nguyen B., Nagai K., Duffield A.S., Gamper C., Small D. (2016). Dnmt3a deletion cooperates with the Flt3/ITD mutation to drive leukemogenesis in a murine model. Oncotarget.

[24] Kats L.M., Reschke M., Taulli R., Pozdnyakova O., Burgess K., Bhargava P., Straley K., Karnik R., Meissner A., Small D. (2014). Proto-oncogenic role of mutant IDH2 in leukemia initiation and maintenance. Cell Stem Cell.

[25] Chen C., Liu Y., Lu C., Cross J.R., Morris J.P.t., Shroff A.S., Ward P.S., Bradner J.E., Thompson C., Lowe S.W. (2013). Cancer-associated IDH2 mutants drive an acute myeloid leukemia that is susceptible to Brd4 inhibition. Genes Dev..

[26] Annesley C.E., Rabik C., Duffield A.S., Rau R.E., Magoon D., Li L., Huff V., Small D., Loeb D.M., Brown P. (2018). Knock-in of the Wt1 R394W mutation causes MDS and cooperates with Flt3/ITD to drive aggressive myeloid neoplasms in mice. Oncotarget.

[27] Pronier E., Bowman R.L., Ahn J., Glass J., Kandoth C., Merlinsky T.R., Whitfield J.T., Durham B.H., Gruet A., Hanasoge Somasundara A.V. (2018). Genetic and epigenetic evolution as a contributor to WT1-mutant leukemogenesis. Blood.

[28] Shih A.H., Jiang Y., Meydan C., Shank K., Pandey S., Barreyro L., Antony-Debre I., Viale A., Socci N., Sun Y. (2015). Mutational cooperativity linked to combinatorial epigenetic gain of function in acute myeloid leukemia. Cancer Cell.

[29] Pacharne S., Dovey O.M., Cooper J.L., Gu M., Friedrich M.J., Rajan S.S., Barenboim M., Collord G., Vijayabaskar M.S., Ponstingl H. (2021). SETBP1 overexpression acts in the place of class-defining mutations to drive FLT3-ITD-mutant AML. Blood Adv..

[30] Supper E., Rudat S., Iyer V., Droop A., Wong K., Spinella J.-F., Thomas P., Sauvageau G., Adams D.J., Wong C.C. (2021). Cut-like homeobox 1 (CUX1) tumor suppressor gene haploinsufficiency induces apoptosis evasion to sustain myeloid leukemia. Nat. Commun..

[31] Thanasopoulou A., Tzankov A., Schwaller J. (2014). Potent co-operation between the NUP98-NSD1 fusion and the FLT3-ITD mutation in acute myeloid leukemia induction. Haematologica.

[32] Greenblatt S., Li L., Slape C., Nguyen B., Novak R., Duffield A., Huso D., Desiderio S., Borowitz M.J., Aplan P. (2012). Knock-in of a FLT3/ITD mutation cooperates with a NUP98-HOXD13 fusion to generate acute myeloid leukemia in a mouse model. Blood.

[33] Stubbs M.C., Kim Y.M., Krivtsov A.V., Wright R.D., Feng Z., Agarwal J., Kung A.L., Armstrong S.A. (2008). MLL-AF9 and FLT3 cooperation in acute myelogenous leukemia: Development of a model for rapid therapeutic assessment. Leukemia.

[34] Schessl C., Rawat V.P., Cusan M., Deshpande A., Kohl T.M., Rosten P.M., Spiekermann K., Humphries R.K., Schnittger S., Kern W. (2005). The AML1-ETO fusion gene and the FLT3 length mutation collaborate in inducing acute leukemia in mice. J. Clin. Investig..

[35] Xiang Z., Kreisel F., Cain J., Colson A., Tomasson M.H. (2007). Neoplasia driven by mutant c-KIT is mediated by intracellular, not plasma membrane, receptor signaling. Mol. Cell Biol..

[36] Wang Y.Y., Zhao L.J., Wu C.F., Liu P., Shi L., Liang Y., Xiong S.M., Mi J.Q., Chen Z., Ren R. (2011). C-KIT mutation cooperates with full-length AML1-ETO to induce acute myeloid leukemia in mice. Proc. Natl. Acad. Sci. USA.

[37] Di Siena S., Gimmelli R., Nori S.L., Barbagallo F., Campolo F., Dolci S., Rossi P., Venneri M.A., Giannetta E., Gianfrilli D. (2016). Activated c-Kit receptor in the heart promotes cardiac repair and regeneration after injury. Cell Death Dis..

[38] Zhao S., Zhang Y., Sha K., Tang Q., Yang X., Yu C., Liu Z., Sun W., Cai L., Xu C. (2014). KRAS (G12D) cooperates with AML1/ETO to initiate a mouse model mimicking human acute myeloid leukemia. Cell Physiol. Biochem..

[39] Braun B.S., Tuveson D.A., Kong N., Le D.T., Kogan S.C., Rozmus J., Le Beau M.M., Jacks T.E., Shannon K.M. (2004). Somatic activation of oncogenic Kras in hematopoietic cells initiates a rapidly fatal myeloproliferative disorder. Proc. Natl. Acad. Sci. USA.

[40] Chan I.T., Kutok J.L., Williams I.R., Cohen S., Kelly L., Shigematsu H., Johnson L., Akashi K., Tuveson D.A., Jacks T. (2004). Conditional expression of oncogenic K-ras from its endogenous promoter induces a myeloproliferative disease. J. Clin. Investig..

[41] Koera K., Nakamura K., Nakao K., Miyoshi J., Toyoshima K., Hatta T., Otani H., Aiba A., Katsuki M. (1997). K-Ras is essential for the development of the mouse embryo. Oncogene.

[42] Johnson L., Greenbaum D., Cichowski K., Mercer K., Murphy E., Schmitt E., Bronson R.T., Umanoff H., Edelmann W., Kucherlapati R. (1997). K-ras is an essential gene in the mouse with partial functional overlap with N-ras. Genes Dev..

[43] Mohanty S., Jyotsana N., Sharma A., Kloos A., Gabdoulline R., Othman B., Lai C.K., Schottmann R., Mandhania M., Schmoellerl J. (2020). Targeted Inhibition of the NUP98-NSD1 Fusion Oncogene in Acute Myeloid Leukemia. Cancers.

[44] Parikh C., Subrahmanyam R., Ren R. (2006). Oncogenic NRAS rapidly and efficiently induces CMML- and AML-like diseases in mice. Blood.

[45] Kogan S.C., Lagasse E., Atwater S., Bae S.C., Weissman I., Ito Y., Bishop J.M. (1998). The PEBP2betaMYH11 fusion created by Inv(16)(p13;q22) in myeloid leukemia impairs neutrophil maturation and contributes to granulocytic dysplasia. Proc. Natl. Acad. Sci. USA.

[46] MacKenzie K.L., Dolnikov A., Millington M., Shounan Y., Symonds G. (1999). Mutant N-ras induces myeloproliferative disorders and apoptosis in bone marrow repopulated mice. Blood.

[47] Wang J., Liu Y., Li Z., Wang Z., Tan L.X., Ryu M.J., Meline B., Du J., Young K.H., Ranheim E. (2011). Endogenous oncogenic Nras mutation initiates hematopoietic malignancies in a dose- and cell type-dependent manner. Blood.

[48] Umanoff H., Edelmann W., Pellicer A., Kucherlapati R. (1995). The murine N-ras gene is not essential for growth and development. Proc. Natl. Acad. Sci. USA.

[49] Brannan C.I., Perkins A.S., Vogel K.S., Ratner N., Nordlund M.L., Reid S.W., Buchberg A.M., Jenkins N.A., Parada L.F., Copeland N.G. (1994). Targeted disruption of the neurofibromatosis type-1 gene leads to developmental abnormalities in heart and various neural crest-derived tissues. Genes Dev..

[50] Jacks T., Shih T.S., Schmitt E.M., Bronson R.T., Bernards A., Weinberg R.A. (1994). Tumour predisposition in mice heterozygous for a targeted mutation in Nf1. Nat. Genet..

[51] Mohi M.G., Williams I.R., Dearolf C.R., Chan G., Kutok J.L., Cohen S., Morgan K., Boulton C., Shigematsu H., Keilhack H. (2005). Prognostic, therapeutic, and mechanistic implications of a mouse model of leukemia evoked by Shp2 (PTPN11) mutations. Cancer Cell.

[52] Chan G., Kalaitzidis D., Usenko T., Kutok J.L., Yang W., Mohi M.G., Neel B.G. (2009). Leukemogenic Ptpn11 causes fatal myeloproliferative disorder via cell-autonomous effects on multiple stages of hematopoiesis. Blood.

[53] Dong L., Yu W.M., Zheng H., Loh M.L., Bunting S.T., Pauly M., Huang G., Zhou M., Broxmeyer H.E., Scadden D.T. (2016). Leukaemogenic effects of Ptpn11 activating mutations in the stem cell microenvironment. Nature.

[54] Saxton T.M., Henkemeyer M., Gasca S., Shen R., Rossi D.J., Shalaby F., Feng G.S., Pawson T. (1997). Abnormal mesoderm patterning in mouse embryos mutant for the SH2 tyrosine phosphatase Shp-2. EMBO J..

[55] Xu J., Wang Y.Y., Dai Y.J., Zhang W., Zhang W.N., Xiong S.M., Gu Z.H., Wang K.K., Zeng R., Chen Z. (2014). DNMT3A Arg882 mutation drives chronic myelomonocytic leukemia through disturbing gene expression/DNA methylation in hematopoietic cells. Proc. Natl. Acad. Sci. USA.

[56] Dai Y.J., Wang Y.Y., Huang J.Y., Xia L., Shi X.D., Xu J., Lu J., Su X.B., Yang Y., Zhang W.N. (2017). Conditional knockin of Dnmt3a R878H initiates acute myeloid leukemia with mTOR pathway involvement. Proc. Natl. Acad. Sci. USA.

[57] Okano M., Bell D.W., Haber D.A., Li E. (1999). DNA methyltransferases Dnmt3a and Dnmt3b are essential for de novo methylation and mammalian development. Cell.

[58] Guryanova O.A., Lieu Y.K., Garrett-Bakelman F.E., Spitzer B., Glass J.L., Shank K., Martinez A.B., Rivera S.A., Durham B.H., Rapaport F. (2016). Dnmt3a regulates myeloproliferation and liver-specific expansion of hematopoietic stem and progenitor cells. Leukemia.

[59] Li Z., Cai X., Cai C.L., Wang J., Zhang W., Petersen B.E., Yang F.C., Xu M. (2011). Deletion of Tet2 in mice leads to dysregulated hematopoietic stem cells and subsequent development of myeloid malignancies. Blood.

[60] Wang J., Miao Z., Jiang Y., Zou P., Li W., Tang X., Lv Y., Xing D., Chen S., Yang F. (2020). Erratum: Characteristics of myeloid sarcoma in mice and patients with TET2 deficiency. Oncol. Lett..

[61] Moran-Crusio K., Reavie L., Shih A., Abdel-Wahab O., Ndiaye-Lobry D., Lobry C., Figueroa M.E., Vasanthakumar A., Patel J., Zhao X. (2011). Tet2 loss leads to increased hematopoietic stem cell self-renewal and myeloid transformation. Cancer Cell.

[62] Chaturvedi A., Araujo Cruz M.M., Jyotsana N., Sharma A., Yun H., Gorlich K., Wichmann M., Schwarzer A., Preller M., Thol F. (2013). Mutant IDH1 promotes leukemogenesis in vivo and can be specifically targeted in human AML. Blood.

[63] Sasaki M., Knobbe C.B., Munger J.C., Lind E.F., Brenner D., Brustle A., Harris I.S., Holmes R., Wakeham A., Haight J. (2012). IDH1(R132H) mutation increases murine haematopoietic progenitors and alters epigenetics. Nature.

[64] Itsumi M., Inoue S., Elia A.J., Murakami K., Sasaki M., Lind E.F., Brenner D., Harris I.S., Chio I.I., Afzal S. (2015). Idh1 protects murine hepatocytes from endotoxin-induced oxidative stress by regulating the intracellular NADP(+)/NADPH ratio. Cell Death Differ..

[65] McKenney A.S., Lau A.N., Somasundara A.V.H., Spitzer B., Intlekofer A.M., Ahn J., Shank K., Rapaport F.T., Patel M.A., Papalexi E. (2018). JAK2/IDH-mutant-driven myeloproliferative neoplasm is sensitive to combined targeted inhibition. J. Clin. Investig..

[66] Shih A.H., Meydan C., Shank K., Garrett-Bakelman F.E., Ward P.S., Intlekofer A.M., Nazir A., Stein E.M., Knapp K., Glass J. (2017). Combination Targeted Therapy to Disrupt Aberrant Oncogenic Signaling and Reverse Epigenetic Dysfunction in IDH2- and TET2-Mutant Acute Myeloid Leukemia. Cancer Discov..

[67] White K., Kim M.-J., Han C., Park H.-J., Ding D., Boyd K., Walker L., Linser P., Meneses Z., Slade C. (2018). Loss of IDH2 Accelerates Age-related Hearing Loss in Male Mice. Sci. Rep..

[68] Herrera-Merchan A., Arranz L., Ligos J.M., de Molina A., Dominguez O., Gonzalez S. (2012). Ectopic expression of the histone methyltransferase Ezh2 in haematopoietic stem cells causes myeloproliferative disease. Nat. Commun..

[69] Mochizuki-Kashio M., Mishima Y., Miyagi S., Negishi M., Saraya A., Konuma T., Shinga J., Koseki H., Iwama A. (2011). Dependency on the polycomb gene Ezh2 distinguishes fetal from adult hematopoietic stem cells. Blood.

[70] Mochizuki-Kashio M., Aoyama K., Sashida G., Oshima M., Tomioka T., Muto T., Wang C., Iwama A. (2015). Ezh2 loss in hematopoietic stem cells predisposes mice to develop heterogeneous malignancies in an Ezh1-dependent manner. Blood.

[71] Inoue D., Kitaura J., Togami K., Nishimura K., Enomoto Y., Uchida T., Kagiyama Y., Kawabata K.C., Nakahara F., Izawa K. (2013). Myelodysplastic syndromes are induced by histone methylation–altering ASXL1 mutations. J. Clin. Investig..

[72] Yang H., Kurtenbach S., Guo Y., Lohse I., Durante M.A., Li J., Li Z., Al-Ali H., Li L., Chen Z. (2018). Gain of function of ASXL1 truncating protein in the pathogenesis of myeloid malignancies. Blood.

[73] Hsu Y.C., Chiu Y.C., Lin C.C., Kuo Y.Y., Hou H.A., Tzeng Y.S., Kao C.J., Chuang P.H., Tseng M.H., Hsiao T.H. (2017). The distinct biological implications of Asxl1 mutation and its roles in leukemogenesis revealed by a knock-in mouse model. J. Hematol. Oncol..

[74] Uni M., Masamoto Y., Sato T., Kamikubo Y., Arai S., Hara E., Kurokawa M. (2019). Modeling ASXL1 mutation revealed impaired hematopoiesis caused by depression of p16Ink4a through aberrant PRC1-mediated histone modification. Leukemia.

[75] Wang J., Li Z., He Y., Pan F., Chen S., Rhodes S., Nguyen L., Yuan J., Jiang L., Yang X. (2014). Loss of Asxl1 leads to myelodysplastic syndrome-like disease in mice. Blood.

[76] Farber C.R., Bennett B.J., Orozco L., Zou W., Lira A., Kostem E., Kang H.M., Furlotte N., Berberyan A., Ghazalpour A. (2011). Mouse genome-wide association and systems genetics identify Asxl2 as a regulator of bone mineral density and osteoclastogenesis. PLoS Genet..

[77] Vikas M., Lin H., Norimichi H., Weoi Woon T., Anand M., Qiao-Yang S., Ling-Wen D., Hazimah Binte Mohd N., Su Lin L., Pavithra S. (2018). ASXL2 regulates hematopoiesis in mice and its deficiency promotes myeloid expansion. Haematologica.

[78] Li J., He F., Zhang P., Chen S., Shi H., Sun Y., Guo Y., Yang H., Man N., Greenblatt S. (2017). Loss of Asxl2 leads to myeloid malignancies in mice. Nat. Commun..

[79] Cheng K., Sportoletti P., Ito K., Clohessy J.G., Teruya-Feldstein J., Kutok J.L., Pandolfi P.P. (2010). The cytoplasmic NPM mutant induces myeloproliferation in a transgenic mouse model. Blood.

[80] Chou S.H., Ko B.S., Chiou J.S., Hsu Y.C., Tsai M.H., Chiu Y.C., Yu I.S., Lin S.W., Hou H.A., Kuo Y.Y. (2012). A knock-in Npm1 mutation in mice results in myeloproliferation and implies a perturbation in hematopoietic microenvironment. PLoS ONE.

[81] Vassiliou G.S., Cooper J.L., Rad R., Li J., Rice S., Uren A., Rad L., Ellis P., Andrews R., Banerjee R. (2011). Mutant nucleophosmin and cooperating pathways drive leukemia initiation and progression in mice. Nat. Genet..

[82] Mallardo M., Caronno A., Pruneri G., Raviele P.R., Viale A., Pelicci P.G., Colombo E. (2013). NPMc+ and FLT3_ITD mutations cooperate in inducing acute leukaemia in a novel mouse model. Leukemia.

[83] Grisendi S., Bernardi R., Rossi M., Cheng K., Khandker L., Manova K., Pandolfi P.P. (2005). Role of nucleophosmin in embryonic development and tumorigenesis. Nature.

[84] Sportoletti P., Grisendi S., Majid S.M., Cheng K., Clohessy J.G., Viale A., Teruya-Feldstein J., Pandolfi P.P. (2008). Npm1 is a haploinsufficient suppressor of myeloid and lymphoid malignancies in the mouse. Blood.

[85] Bereshchenko O., Mancini E., Moore S., Bilbao D., Mansson R., Luc S., Grover A., Jacobsen S.E., Bryder D., Nerlov C. (2009). Hematopoietic stem cell expansion precedes the generation of committed myeloid leukemia-initiating cells in C/EBPalpha mutant AML. Cancer Cell.

[86] Wang N.D., Finegold M.J., Bradley A., Ou C.N., Abdelsayed S.V., Wilde M.D., Taylor L.R., Wilson D.R., Darlington G.J. (1995). Impaired energy homeostasis in C/EBP alpha knockout mice. Science.

[87] Kirstetter P., Schuster M.B., Bereshchenko O., Moore S., Dvinge H., Kurz E., Theilgaard-Monch K., Mansson R., Pedersen T.A., Pabst T. (2008). Modeling of C/EBPalpha mutant acute myeloid leukemia reveals a common expression signature of committed myeloid leukemia-initiating cells. Cancer Cell.

[88] Watanabe-Okochi N., Kitaura J., Ono R., Harada H., Harada Y., Komeno Y., Nakajima H., Nosaka T., Inaba T., Kitamura T. (2008). AML1 mutations induced MDS and MDS/AML in a mouse BMT model. Blood.

[89] Okuda T., van Deursen J., Hiebert S.W., Grosveld G., Downing J.R. (1996). AML1, the target of multiple chromosomal translocations in human leukemia, is essential for normal fetal liver hematopoiesis. Cell.

[90] Luo H., Li Q., O’Neal J., Kreisel F., Le Beau M.M., Tomasson M.H. (2005). c-Myc rapidly induces acute myeloid leukemia in mice without evidence of lymphoma-associated antiapoptotic mutations. Blood.

[91] Kawagoe H., Kandilci A., Kranenburg T.A., Grosveld G.C. (2007). Overexpression of N-Myc rapidly causes acute myeloid leukemia in mice. Cancer Res..

[92] Davis A.C., Wims M., Spotts G.D., Hann S.R., Bradley A. (1993). A null c-myc mutation causes lethality before 10.5 days of gestation in homozygotes and reduced fertility in heterozygous female mice. Genes Dev..

[93] Tanaka T., Nakajima-Takagi Y., Aoyama K., Tara S., Oshima M., Saraya A., Koide S., Si S., Manabe I., Sanada M. (2017). Internal deletion of BCOR reveals a tumor suppressor function for BCOR in T lymphocyte malignancies. J. Exp. Med..

[94] Tara S., Isshiki Y., Nakajima-Takagi Y., Oshima M., Aoyama K., Tanaka T., Shinoda D., Koide S., Saraya A., Miyagi S. (2018). Bcor insufficiency promotes initiation and progression of myelodysplastic syndrome. Blood.

[95] Wamstad J.A., Corcoran C.M., Keating A.M., Bardwell V.J. (2008). Role of the transcriptional corepressor Bcor in embryonic stem cell differentiation and early embryonic development. PLoS ONE.

[96] Cadieux C., Fournier S., Peterson A.C., Bédard C., Bedell B.J., Nepveu A. (2006). Transgenic mice expressing the p75 CCAAT-displacement protein/Cut homeobox isoform develop a myeloproliferative disease-like myeloid leukemia. Cancer Res..

[97] Luong M.X., van der Meijden C.M., Xing D., Hesselton R., Monuki E.S., Jones S.N., Lian J.B., Stein J.L., Stein G.S., Neufeld E.J. (2002). Genetic ablation of the CDP/Cux protein C terminus results in hair cycle defects and reduced male fertility. Mol. Cell Biol..

[98] Vishwakarma B.A., Nguyen N., Makishima H., Hosono N., Gudmundsson K.O., Negi V., Oakley K., Han Y., Przychodzen B., Maciejewski J.P. (2016). Runx1 repression by histone deacetylation is critical for Setbp1-induced mouse myeloid leukemia development. Leukemia.

[99] Inoue D., Kitaura J., Matsui H., Hou H.A., Chou W.C., Nagamachi A., Kawabata K.C., Togami K., Nagase R., Horikawa S. (2015). SETBP1 mutations drive leukemic transformation in ASXL1-mutated MDS. Leukemia.

[100] Hsu Y.C., Chen T.C., Lin C.C., Yuan C.T., Hsu C.L., Hou H.A., Kao C.J., Chuang P.H., Chen Y.R., Chou W.C. (2019). Phf6-null hematopoietic stem cells have enhanced self-renewal capacity and oncogenic potentials. Blood Adv..

[101] McRae H.M., Garnham A.L., Hu Y., Witkowski M.T., Corbett M.A., Dixon M.P., May R.E., Sheikh B.N., Chiang W., Kueh A.J. (2019). PHF6 regulates hematopoietic stem and progenitor cells and its loss synergizes with expression of TLX3 to cause leukemia. Blood.

[102] Kreidberg J.A., Sariola H., Loring J.M., Maeda M., Pelletier J., Housman D., Jaenisch R. (1993). WT-1 is required for early kidney development. Cell.

[103] Loizou E., Banito A., Livshits G., Ho Y.J., Koche R.P., Sanchez-Rivera F.J., Mayle A., Chen C.C., Kinalis S., Bagger F.O. (2019). A Gain-of-Function p53-Mutant Oncogene Promotes Cell Fate Plasticity and Myeloid Leukemia through the Pluripotency Factor FOXH1. Cancer Discov..

[104] Hanel W., Marchenko N., Xu S., Yu S.X., Weng W., Moll U. (2013). Two hot spot mutant p53 mouse models display differential gain of function in tumorigenesis. Cell Death Differ..

[105] Donehower L.A., Harvey M., Slagle B.L., McArthur M.J., Montgomery C.A., Butel J.S., Bradley A. (1992). Mice deficient for p53 are developmentally normal but susceptible to spontaneous tumours. Nature.

[106] Jacks T., Remington L., Williams B.O., Schmitt E.M., Halachmi S., Bronson R.T., Weinberg R.A. (1994). Tumor spectrum analysis in p53-mutant mice. Curr. Biol..

[107] Komeno Y., Huang Y.J., Qiu J., Lin L., Xu Y., Zhou Y., Chen L., Monterroza D.D., Li H., DeKelver R.C. (2015). SRSF2 Is Essential for Hematopoiesis, and Its Myelodysplastic Syndrome-Related Mutations Dysregulate Alternative Pre-mRNA Splicing. Mol. Cell Biol..

[108] Kim E., Ilagan J.O., Liang Y., Daubner G.M., Lee S.C., Ramakrishnan A., Li Y., Chung Y.R., Micol J.B., Murphy M.E. (2015). SRSF2 Mutations Contribute to Myelodysplasia by Mutant-Specific Effects on Exon Recognition. Cancer Cell.

[109] Kon A., Yamazaki S., Nannya Y., Kataoka K., Ota Y., Nakagawa M.M., Yoshida K., Shiozawa Y., Morita M., Yoshizato T. (2018). Physiological Srsf2 P95H expression causes impaired hematopoietic stem cell functions and aberrant RNA splicing in mice. Blood.

[110] Shirai C.L., Ley J.N., White B.S., Kim S., Tibbitts J., Shao J., Ndonwi M., Wadugu B., Duncavage E.J., Okeyo-Owuor T. (2015). Mutant U2AF1 Expression Alters Hematopoiesis and Pre-mRNA Splicing In Vivo. Cancer Cell.

[111] Fei D.L., Zhen T., Durham B., Ferrarone J., Zhang T., Garrett L., Yoshimi A., Abdel-Wahab O., Bradley R.K., Liu P. (2018). Impaired hematopoiesis and leukemia development in mice with a conditional knock-in allele of a mutant splicing factor gene U2af1. Proc. Natl. Acad. Sci. USA.

[112] Wadugu B.A., Heard A., Srivatsan S.N., Alberti M.O., Ndonwi M., Grieb S., Bradley J., Shao J., Ahmed T., Shirai C.L. (2020). *U2AF1* is a haplo-essential gene required for cancer cell survival. bioRxiv.

[113] Mupo A., Seiler M., Sathiaseelan V., Pance A., Yang Y., Agrawal A.A., Iorio F., Bautista R., Pacharne S., Tzelepis K. (2017). Hemopoietic-specific Sf3b1-K700E knock-in mice display the splicing defect seen in human MDS but develop anemia without ring sideroblasts. Leukemia.

[114] Obeng E.A., Chappell R.J., Seiler M., Chen M.C., Campagna D.R., Schmidt P.J., Schneider R.K., Lord A.M., Wang L., Gambe R.G. (2016). Physiologic Expression of Sf3b1(K700E) Causes Impaired Erythropoiesis, Aberrant Splicing, and Sensitivity to Therapeutic Spliceosome Modulation. Cancer Cell.

[115] Visconte V., Tabarroki A., Zhang L., Parker Y., Hasrouni E., Mahfouz R., Isono K., Koseki H., Sekeres M.A., Saunthararajah Y. (2014). Splicing factor 3b subunit 1 (Sf3b1) haploinsufficient mice display features of low risk Myelodysplastic syndromes with ring sideroblasts. J. Hematol. Oncol..

[116] Isono K., Mizutani-Koseki Y., Komori T., Schmidt-Zachmann M.S., Koseki H. (2005). Mammalian polycomb-mediated repression of Hox genes requires the essential spliceosomal protein Sf3b1. Genes Dev..

[117] Xu H., Balakrishnan K., Malaterre J., Beasley M., Yan Y., Essers J., Appeldoorn E., Tomaszewski J.M., Vazquez M., Verschoor S. (2010). Rad21-cohesin haploinsufficiency impedes DNA repair and enhances gastrointestinal radiosensitivity in mice. PLoS ONE.

[118] Remeseiro S., Cuadrado A., Carretero M., Martinez P., Drosopoulos W.C., Canamero M., Schildkraut C.L., Blasco M.A., Losada A. (2012). Cohesin-SA1 deficiency drives aneuploidy and tumourigenesis in mice due to impaired replication of telomeres. EMBO J..

[119] De Koninck M., Lapi E., Badia-Careaga C., Cossio I., Gimenez-Llorente D., Rodriguez-Corsino M., Andrada E., Hidalgo A., Manzanares M., Real F.X. (2020). Essential Roles of Cohesin STAG2 in Mouse Embryonic Development and Adult Tissue Homeostasis. Cell Rep..

[120] Wang T., Glover B., Hadwiger G., Miller C.A., di Martino O., Welch J.S. (2019). Smc3 is required for mouse embryonic and adult hematopoiesis. Exp. Hematol..

[121] Rau R., Magoon D., Greenblatt S., Li L., Annesley C., Duffield A.S., Huso D., McIntyre E., Clohessy J.G., Reschke M. (2014). NPMc+ cooperates with Flt3/ITD mutations to cause acute leukemia recapitulating human disease. Exp. Hematol..

[122] Mupo A., Celani L., Dovey O., Cooper J.L., Grove C., Rad R., Sportoletti P., Falini B., Bradley A., Vassiliou G.S. (2013). A powerful molecular synergy between mutant Nucleophosmin and Flt3-ITD drives acute myeloid leukemia in mice. Leukemia.

[123] Chang Y.I., You X., Kong G., Ranheim E.A., Wang J., Du J., Liu Y., Zhou Y., Ryu M.J., Zhang J. (2015). Loss of Dnmt3a and endogenous Kras(G12D/+) cooperate to regulate hematopoietic stem and progenitor cell functions in leukemogenesis. Leukemia.

[124] Zhao Z., Zuber J., Diaz-Flores E., Lintault L., Kogan S.C., Shannon K., Lowe S.W. (2010). p53 loss promotes acute myeloid leukemia by enabling aberrant self-renewal. Genes Dev..

[125] Cutts B.A., Sjogren A.K., Andersson K.M., Wahlstrom A.M., Karlsson C., Swolin B., Bergo M.O. (2009). Nf1 deficiency cooperates with oncogenic K-RAS to induce acute myeloid leukemia in mice. Blood.

[126] Kelly M.J., So J., Rogers A.J., Gregory G., Li J., Zethoven M., Gearhart M.D., Bardwell V.J., Johnstone R.W., Vervoort S.J. (2019). Bcor loss perturbs myeloid differentiation and promotes leukaemogenesis. Nat. Commun..

[127] Zhang J., Kong G., Rajagopalan A., Lu L., Song J., Hussaini M., Zhang X., Ranheim E.A., Liu Y., Wang J. (2017). p53−/− synergizes with enhanced NrasG12D signaling to transform megakaryocyte-erythroid progenitors in acute myeloid leukemia. Blood.

[128] Shi X., Yang Y., Shang S., Wu S., Zhang W., Peng L., Huang T., Zhang R., Ren R., Mi J. (2019). Cooperation of Dnmt3a R878H with Nras G12D promotes leukemogenesis in knock-in mice: A pilot study. BMC Cancer.

[129] Dovey O.M., Cooper J.L., Mupo A., Grove C.S., Lynn C., Conte N., Andrews R.M., Pacharne S., Tzelepis K., Vijayabaskar M.S. (2017). Molecular synergy underlies the co-occurrence patterns and phenotype of NPM1-mutant acute myeloid leukemia. Blood.

[130] Gu Z., Liu Y., Cai F., Patrick M., Zmajkovic J., Cao H., Zhang Y., Tasdogan A., Chen M., Qi L. (2019). Loss of EZH2 Reprograms BCAA Metabolism to Drive Leukemic Transformation. Cancer Discov..

[131] Zhang P., He F., Bai J., Yamamoto S., Chen S., Zhang L., Sheng M., Zhang L., Guo Y., Man N. (2018). Chromatin regulator Asxl1 loss and Nf1 haploinsufficiency cooperate to accelerate myeloid malignancy. J. Clin. Investig..

[132] Lu R., Wang P., Parton T., Zhou Y., Chrysovergis K., Rockowitz S., Chen W.Y., Abdel-Wahab O., Wade P.A., Zheng D. (2016). Epigenetic Perturbations by Arg882-Mutated DNMT3A Potentiate Aberrant Stem Cell Gene-Expression Program and Acute Leukemia Development. Cancer Cell.

[133] Zhang X., Wang X., Wang X.Q.D., Su J., Putluri N., Zhou T., Qu Y., Jeong M., Guzman A., Rosas C. (2020). Dnmt3a loss and Idh2 neomorphic mutations mutually potentiate malignant hematopoiesis. Blood.

[134] Loberg M.A., Bell R.K., Goodwin L.O., Eudy E., Miles L.A., SanMiguel J.M., Young K., Bergstrom D.E., Levine R.L., Schneider R.K. (2019). Sequentially inducible mouse models reveal that Npm1 mutation causes malignant transformation of Dnmt3a-mutant clonal hematopoiesis. Leukemia.

[135] Sportoletti P., Sorcini D., Guzman A.G., Reyes J.M., Stella A., Marra A., Sartori S., Brunetti L., Rossi R., Papa B.D. (2020). Bcor deficiency perturbs erythro-megakaryopoiesis and cooperates with Dnmt3a loss in acute erythroid leukemia onset in mice. Leukemia.

[136] D’Altri T., Wilhelmson A.S., Schuster M.B., Wenzel A., Kalvisa A., Pundhir S., Meldgaard Hansen A., Porse B.T. (2021). The ASXL1-G643W variant accelerates the development of CEBPA mutant acute myeloid leukemia. Haematologica.

[137] Edling C.E., Hallberg B. (2007). c-Kit—A hematopoietic cell essential receptor tyrosine kinase. Int. J. Biochem. Cell Biol..

[138] Beghini A., Peterlongo P., Ripamonti C.B., Larizza L., Cairoli R., Morra E., Mecucci C. (2000). C-kit mutations in core binding factor leukemias. Blood.

[139] Paschka P., Marcucci G., Ruppert A.S., Mrózek K., Chen H., Kittles R.A., Vukosavljevic T., Perrotti D., Vardiman J.W., Carroll A.J. (2006). Adverse prognostic significance of KIT mutations in adult acute myeloid leukemia with inv(16) and t(8;21): A Cancer and Leukemia Group B Study. J. Clin. Oncol. Off. J. Am. Soc. Clin. Oncol..

[140] Ishikawa Y., Kawashima N., Atsuta Y., Sugiura I., Sawa M., Dobashi N., Yokoyama H., Doki N., Tomita A., Kiguchi T. (2020). Prospective evaluation of prognostic impact of KIT mutations on acute myeloid leukemia with RUNX1-RUNX1T1 and CBFB-MYH11. Blood Adv..

[141] Nick H.J., Kim H.G., Chang C.W., Harris K.W., Reddy V., Klug C.A. (2012). Distinct classes of c-Kit-activating mutations differ in their ability to promote RUNX1-ETO-associated acute myeloid leukemia. Blood.

[142] Müller A.M., Duque J., Shizuru J.A., Lübbert M. (2008). Complementing mutations in core binding factor leukemias: From mouse models to clinical applications. Oncogene.

[143] Zhao L., Melenhorst J.J., Alemu L., Kirby M., Anderson S., Kench M., Hoogstraten-Miller S., Brinster L., Kamikubo Y., Gilliland D.G. (2012). KIT with D816 mutations cooperates with CBFB-MYH11 for leukemogenesis in mice. Blood.

[144] Ward A.F., Braun B.S., Shannon K.M. (2012). Targeting oncogenic Ras signaling in hematologic malignancies. Blood.

[145] Lowy D.R., Willumsen B.M. (1993). Function and regulation of ras. Annu. Rev. Biochem..

[146] Bowen D.T., Frew M.E., Hills R., Gale R.E., Wheatley K., Groves M.J., Langabeer S.E., Kottaridis P.D., Moorman A.V., Burnett A.K. (2005). RAS mutation in acute myeloid leukemia is associated with distinct cytogenetic subgroups but does not influence outcome in patients younger than 60 years. Blood.

[147] Bacher U., Haferlach T., Schoch C., Kern W., Schnittger S. (2006). Implications of NRAS mutations in AML: A study of 2502 patients. Blood.

[148] Wang S., Wu Z., Li T., Li Y., Wang W., Hao Q., Xie X., Wan D., Jiang Z., Wang C. (2020). Mutational spectrum and prognosis in NRAS-mutated acute myeloid leukemia. Sci. Rep..

[149] Li Q., Haigis K.M., McDaniel A., Harding-Theobald E., Kogan S.C., Akagi K., Wong J.C., Braun B.S., Wolff L., Jacks T. (2011). Hematopoiesis and leukemogenesis in mice expressing oncogenic NrasG12D from the endogenous locus. Blood.

[150] Wang J., Liu Y., Li Z., Du J., Ryu M.J., Taylor P.R., Fleming M.D., Young K.H., Pitot H., Zhang J. (2010). Endogenous oncogenic Nras mutation promotes aberrant GM-CSF signaling in granulocytic/monocytic precursors in a murine model of chronic myelomonocytic leukemia. Blood.

[151] Serrano M., Lin A.W., McCurrach M.E., Beach D., Lowe S.W. (1997). Oncogenic ras provokes premature cell senescence associated with accumulation of p53 and p16INK4a. Cell.

[152] Tuveson D.A., Shaw A.T., Willis N.A., Silver D.P., Jackson E.L., Chang S., Mercer K.L., Grochow R., Hock H., Crowley D. (2004). Endogenous oncogenic K-ras(G12D) stimulates proliferation and widespread neoplastic and developmental defects. Cancer Cell.

[153] Schmoellerl J., Barbosa I.A.M., Eder T., Brandstoetter T., Schmidt L., Maurer B., Troester S., Pham H.T.T., Sagarajit M., Ebner J. (2020). CDK6 is an essential direct target of NUP98 fusion proteins in acute myeloid leukemia. Blood.

[154] Kim W.I., Matise I., Diers M.D., Largaespada D.A. (2009). RAS oncogene suppression induces apoptosis followed by more differentiated and less myelosuppressive disease upon relapse of acute myeloid leukemia. Blood.

[155] Zuber J., Radtke I., Pardee T.S., Zhao Z., Rappaport A.R., Luo W., McCurrach M.E., Yang M.M., Dolan M.E., Kogan S.C. (2009). Mouse models of human AML accurately predict chemotherapy response. Genes Dev..

[156] Philpott C., Tovell H., Frayling I.M., Cooper D.N., Upadhyaya M. (2017). The NF1 somatic mutational landscape in sporadic human cancers. Hum. Genom..

[157] Eisfeld A.K., Kohlschmidt J., Mrózek K., Mims A., Walker C.J., Blachly J.S., Nicolet D., Orwick S., Maharry S.E., Carroll A.J. (2018). NF1 mutations are recurrent in adult acute myeloid leukemia and confer poor outcome. Leukemia.

[158] Le D.T., Kong N., Zhu Y., Lauchle J.O., Aiyigari A., Braun B.S., Wang E., Kogan S.C., Le Beau M.M., Parada L. (2004). Somatic inactivation of Nf1 in hematopoietic cells results in a progressive myeloproliferative disorder. Blood.

[159] Kim A., Morgan K., Hasz D.E., Wiesner S.M., Lauchle J.O., Geurts J.L., Diers M.D., Le D.T., Kogan S.C., Parada L.F. (2007). Beta common receptor inactivation attenuates myeloproliferative disease in Nf1 mutant mice. Blood.

[160] Chan R.J., Feng G.S. (2007). PTPN11 is the first identified proto-oncogene that encodes a tyrosine phosphatase. Blood.

[161] Grossmann K.S., Rosário M., Birchmeier C., Birchmeier W. (2010). The tyrosine phosphatase Shp2 in development and cancer. Adv. Cancer Res..

[162] Pandey R., Saxena M., Kapur R. (2017). Role of SHP2 in hematopoiesis and leukemogenesis. Curr. Opin. Hematol..

[163] Alfayez M., Issa G.C., Patel K.P., Wang F., Wang X., Short N.J., Cortes J.E., Kadia T., Ravandi F., Pierce S. (2021). The Clinical impact of PTPN11 mutations in adults with acute myeloid leukemia. Leukemia.

[164] Chan G., Cheung L.S., Yang W., Milyavsky M., Sanders A.D., Gu S., Hong W.X., Liu A.X., Wang X., Barbara M. (2011). Essential role for Ptpn11 in survival of hematopoietic stem and progenitor cells. Blood.

[165] Tarnawsky S.P., Yu W.M., Qu C.K., Chan R.J., Yoder M.C. (2018). Hematopoietic-restricted Ptpn11E76K reveals indolent MPN progression in mice. Oncotarget.

[166] Xu D., Liu X., Yu W.M., Meyerson H.J., Guo C., Gerson S.L., Qu C.K. (2011). Non-lineage/stage-restricted effects of a gain-of-function mutation in tyrosine phosphatase Ptpn11 (Shp2) on malignant transformation of hematopoietic cells. J. Exp. Med..

[167] Chen L., Chen W., Mysliwski M., Serio J., Ropa J., Abulwerdi F.A., Chan R.J., Patel J.P., Tallman M.S., Paietta E. (2015). Mutated Ptpn11 alters leukemic stem cell frequency and reduces the sensitivity of acute myeloid leukemia cells to Mcl1 inhibition. Leukemia.

[168] Fu J.F., Liang S.T., Huang Y.J., Liang K.H., Yen T.H., Liang D.C., Shih L.Y. (2017). Cooperation of MLL/AF10(OM-LZ) with PTPN11 activating mutation induced monocytic leukemia with a shorter latency in a mouse bone marrow transplantation model. Int. J. Cancer.

[169] Brunetti L., Gundry M.C., Goodell M.A. (2017). DNMT3A in Leukemia. Cold Spring Harb. Perspect. Med..

[170] Wouters B.J., Delwel R. (2016). Epigenetics and approaches to targeted epigenetic therapy in acute myeloid leukemia. Blood.

[171] Ley T.J., Miller C., Ding L., Raphael B.J., Mungall A.J., Robertson A., Hoadley K., Triche T.J., Laird P.W., Baty J.D. (2013). Genomic and epigenomic landscapes of adult de novo acute myeloid leukemia. N. Engl. J. Med..

[172] Challen G.A., Sun D., Jeong M., Luo M., Jelinek J., Berg J.S., Bock C., Vasanthakumar A., Gu H., Xi Y. (2012). Dnmt3a is essential for hematopoietic stem cell differentiation. Nat. Genet..

[173] Mayle A., Yang L., Rodriguez B., Zhou T., Chang E., Curry C.V., Challen G.A., Li W., Wheeler D., Rebel V.I. (2015). Dnmt3a loss predisposes murine hematopoietic stem cells to malignant transformation. Blood.

[174] Yang L., Rodriguez B., Mayle A., Park H.J., Lin X., Luo M., Jeong M., Curry C.V., Kim S.B., Ruau D. (2016). DNMT3A Loss Drives Enhancer Hypomethylation in FLT3-ITD-Associated Leukemias. Cancer Cell.

[175] Celik H., Mallaney C., Kothari A., Ostrander E.L., Eultgen E., Martens A., Miller C.A., Hundal J., Klco J.M., Challen G.A. (2015). Enforced differentiation of Dnmt3a-null bone marrow leads to failure with c-Kit mutations driving leukemic transformation. Blood.

[176] Deng L., Richine B.M., Virts E.L., Jideonwo-Auman V.N., Chan R.J., Kapur R. (2018). Rapid development of myeloproliferative neoplasm in mice with Ptpn11(D61Y) mutation and haploinsufficient for Dnmt3a. Oncotarget.

[177] Tahiliani M., Koh K.P., Shen Y., Pastor W.A., Bandukwala H., Brudno Y., Agarwal S., Iyer L.M., Liu D.R., Aravind L. (2009). Conversion of 5-methylcytosine to 5-hydroxymethylcytosine in mammalian DNA by MLL partner TET1. Science.

[178] Ko M., Huang Y., Jankowska A.M., Pape U.J., Tahiliani M., Bandukwala H.S., An J., Lamperti E.D., Koh K.P., Ganetzky R. (2010). Impaired hydroxylation of 5-methylcytosine in myeloid cancers with mutant TET2. Nature.

[179] Ito S., D’Alessio A.C., Taranova O.V., Hong K., Sowers L.C., Zhang Y. (2010). Role of Tet proteins in 5mC to 5hmC conversion, ES-cell self-renewal and inner cell mass specification. Nature.

[180] Delhommeau F., Dupont S., Della Valle V., James C., Trannoy S., Massé A., Kosmider O., Le Couedic J.P., Robert F., Alberdi A. (2009). Mutation in TET2 in myeloid cancers. N. Engl. J. Med..

[181] Abdel-Wahab O., Mullally A., Hedvat C., Garcia-Manero G., Patel J., Wadleigh M., Malinge S., Yao J., Kilpivaara O., Bhat R. (2009). Genetic characterization of TET1, TET2, and TET3 alterations in myeloid malignancies. Blood.

[182] Pronier E., Almire C., Mokrani H., Vasanthakumar A., Simon A., da Costa Reis Monte Mor B., Massé A., Le Couédic J.P., Pendino F., Carbonne B. (2011). Inhibition of TET2-mediated conversion of 5-methylcytosine to 5-hydroxymethylcytosine disturbs erythroid and granulomonocytic differentiation of human hematopoietic progenitors. Blood.

[183] An J., Gonzalez-Avalos E., Chawla A., Jeong M., Lopez-Moyado I.F., Li W., Goodell M.A., Chavez L., Ko M., Rao A. (2015). Acute loss of TET function results in aggressive myeloid cancer in mice. Nat. Commun..

[184] Shrestha R., Sakata-Yanagimoto M., Maie K., Oshima M., Ishihara M., Suehara Y., Fukumoto K., Nakajima-Takagi Y., Matsui H., Kato T. (2020). Molecular pathogenesis of progression to myeloid leukemia from TET-insufficient status. Blood Adv..

[185] Kunimoto H., Meydan C., Nazir A., Whitfield J., Shank K., Rapaport F., Maher R., Pronier E., Meyer S.C., Garrett-Bakelman F.E. (2018). Cooperative Epigenetic Remodeling by TET2 Loss and NRAS Mutation Drives Myeloid Transformation and MEK Inhibitor Sensitivity. Cancer Cell.

[186] Muto T., Sashida G., Oshima M., Wendt G.R., Mochizuki-Kashio M., Nagata Y., Sanada M., Miyagi S., Saraya A., Kamio A. (2013). Concurrent loss of Ezh2 and Tet2 cooperates in the pathogenesis of myelodysplastic disorders. J. Exp. Med..

[187] Dang L., Yen K., Attar E.C. (2016). IDH mutations in cancer and progress toward development of targeted therapeutics. Ann. Oncol. Off. J. Eur. Soc. Med. Oncol..

[188] Montalban-Bravo G., DiNardo C.D. (2018). The role of IDH mutations in acute myeloid leukemia. Future Oncol..

[189] Xu W., Yang H., Liu Y., Yang Y., Wang P., Kim S.H., Ito S., Yang C., Wang P., Xiao M.T. (2011). Oncometabolite 2-hydroxyglutarate is a competitive inhibitor of α-ketoglutarate-dependent dioxygenases. Cancer Cell.

[190] Figueroa M.E., Abdel-Wahab O., Lu C., Ward P.S., Patel J., Shih A., Li Y., Bhagwat N., Vasanthakumar A., Fernandez H.F. (2010). Leukemic IDH1 and IDH2 mutations result in a hypermethylation phenotype, disrupt TET2 function, and impair hematopoietic differentiation. Cancer Cell.

[191] Kattih B., Shirvani A., Klement P., Garrido A.M., Gabdoulline R., Liebich A., Brandes M., Chaturvedi A., Seeger T., Thol F. (2020). IDH1/2 mutations in acute myeloid leukemia patients and risk of coronary artery disease and cardiac dysfunction—a retrospective propensity score analysis. Leukemia.

[192] Chaturvedi A., Araujo Cruz M.M., Jyotsana N., Sharma A., Goparaju R., Schwarzer A., Görlich K., Schottmann R., Struys E.A., Jansen E.E. (2016). Enantiomer-specific and paracrine leukemogenicity of mutant IDH metabolite 2-hydroxyglutarate. Leukemia.

[193] Ogawara Y., Katsumoto T., Aikawa Y., Shima Y., Kagiyama Y., Soga T., Matsunaga H., Seki T., Araki K., Kitabayashi I. (2015). IDH2 and NPM1 Mutations Cooperate to Activate Hoxa9/Meis1 and Hypoxia Pathways in Acute Myeloid Leukemia. Cancer Res..

[194] Safaei S., Baradaran B., Hagh M.F., Alivand M.R., Talebi M., Gharibi T., Solali S. (2018). Double sword role of EZH2 in leukemia. Biomed. Pharmacother. Biomed. Pharmacother..

[195] Sashida G., Harada H., Matsui H., Oshima M., Yui M., Harada Y., Tanaka S., Mochizuki-Kashio M., Wang C., Saraya A. (2014). Ezh2 loss promotes development of myelodysplastic syndrome but attenuates its predisposition to leukaemic transformation. Nat. Commun.

[196] Neff T., Sinha A.U., Kluk M.J., Zhu N., Khattab M.H., Stein L., Xie H., Orkin S.H., Armstrong S.A. (2012). Polycomb repressive complex 2 is required for MLL-AF9 leukemia. Proc. Natl. Acad. Sci. USA.

[197] Tanaka S., Miyagi S., Sashida G., Chiba T., Yuan J., Mochizuki-Kashio M., Suzuki Y., Sugano S., Nakaseko C., Yokote K. (2012). Ezh2 augments leukemogenicity by reinforcing differentiation blockage in acute myeloid leukemia. Blood.

[198] Zhang P., Xu M., Yang F.-C. (2020). The Role of ASXL1/2 and Their Associated Proteins in Malignant Hematopoiesis. Curr. Stem Cell Rep..

[199] Chou W.C., Huang H.H., Hou H.A., Chen C.Y., Tang J.L., Yao M., Tsay W., Ko B.S., Wu S.J., Huang S.Y. (2010). Distinct clinical and biological features of de novo acute myeloid leukemia with additional sex comb-like 1 (ASXL1) mutations. Blood.

[200] Asada S., Fujino T., Goyama S., Kitamura T. (2019). The role of ASXL1 in hematopoiesis and myeloid malignancies. Cell. Mol. Life Sci..

[201] Abdel-Wahab O., Adli M., LaFave L.M., Gao J., Hricik T., Shih A.H., Pandey S., Patel J.P., Chung Y.R., Koche R. (2012). ASXL1 mutations promote myeloid transformation through loss of PRC2-mediated gene repression. Cancer Cell.

[202] Micol J.B., Duployez N., Boissel N., Petit A., Geffroy S., Nibourel O., Lacombe C., Lapillonne H., Etancelin P., Figeac M. (2014). Frequent ASXL2 mutations in acute myeloid leukemia patients with t(8;21)/RUNX1-RUNX1T1 chromosomal translocations. Blood.

[203] Huether R., Dong L., Chen X., Wu G., Parker M., Wei L., Ma J., Edmonson M.N., Hedlund E.K., Rusch M.C. (2014). The landscape of somatic mutations in epigenetic regulators across 1000 paediatric cancer genomes. Nat. Commun..

[204] Micol J.-B., Pastore A., Inoue D., Duployez N., Kim E., Lee S.C.-W., Durham B.H., Chung Y.R., Cho H., Zhang X.J. (2017). ASXL2 is essential for haematopoiesis and acts as a haploinsufficient tumour suppressor in leukemia. Nat. Commun..

[205] Jeong E.G., Lee S.H., Yoo N.J., Lee S.H. (2007). Absence of nucleophosmin 1 (NPM1) gene mutations in common solid cancers. APMIS.

[206] Rau R., Brown P. (2009). Nucleophosmin (NPM1) mutations in adult and childhood acute myeloid leukaemia: Towards definition of a new leukaemia entity. Hematol. Oncol..

[207] Zarka J., Short N.J., Kanagal-Shamanna R., Issa G.C. (2020). Nucleophosmin 1 Mutations in Acute Myeloid Leukemia. Genes.

[208] Falini B., Mecucci C., Tiacci E., Alcalay M., Rosati R., Pasqualucci L., La Starza R., Diverio D., Colombo E., Santucci A. (2005). Cytoplasmic nucleophosmin in acute myelogenous leukemia with a normal karyotype. N. Engl. J. Med..

[209] Falini B., Bolli N., Shan J., Martelli M.P., Liso A., Pucciarini A., Bigerna B., Pasqualucci L., Mannucci R., Rosati R. (2006). Both carboxy-terminus NES motif and mutated tryptophan(s) are crucial for aberrant nuclear export of nucleophosmin leukemic mutants in NPMc+ AML. Blood.

[210] Falini B., Nicoletti I., Martelli M.F., Mecucci C. (2007). Acute myeloid leukemia carrying cytoplasmic/mutated nucleophosmin (NPMc+ AML): Biologic and clinical features. Blood.

[211] Alcalay M., Tiacci E., Bergomas R., Bigerna B., Venturini E., Minardi S.P., Meani N., Diverio D., Bernard L., Tizzoni L. (2005). Acute myeloid leukemia bearing cytoplasmic nucleophosmin (NPMc+ AML) shows a distinct gene expression profile characterized by up-regulation of genes involved in stem-cell maintenance. Blood.

[212] Uckelmann H.J., Kim S.M., Wong E.M., Hatton C., Giovinazzo H., Gadrey J.Y., Krivtsov A.V., Rucker F.G., Dohner K., McGeehan G.M. (2020). Therapeutic targeting of preleukemia cells in a mouse model of NPM1 mutant acute myeloid leukemia. Science.

[213] Dzama M.M., Steiner M., Rausch J., Sasca D., Schönfeld J., Kunz K., Taubert M.C., McGeehan G.M., Chen C.W., Mupo A. (2020). Synergistic targeting of FLT3 mutations in AML via combined menin-MLL and FLT3 inhibition. Blood.

[214] Avellino R., Delwel R. (2017). Expression and regulation of C/EBPα in normal myelopoiesis and in malignant transformation. Blood.

[215] Wouters B.J., Löwenberg B., Erpelinck-Verschueren C.A., van Putten W.L., Valk P.J., Delwel R. (2009). Double CEBPA mutations, but not single CEBPA mutations, define a subgroup of acute myeloid leukemia with a distinctive gene expression profile that is uniquely associated with a favorable outcome. Blood.

[216] Taskesen E., Bullinger L., Corbacioglu A., Sanders M.A., Erpelinck C.A., Wouters B.J., van der Poel-van de Luytgaarde S.C., Damm F., Krauter J., Ganser A. (2011). Prognostic impact, concurrent genetic mutations, and gene expression features of AML with CEBPA mutations in a cohort of 1182 cytogenetically normal AML patients: Further evidence for CEBPA double mutant AML as a distinctive disease entity. Blood.

[217] Leroy H., Roumier C., Huyghe P., Biggio V., Fenaux P., Preudhomme C. (2005). CEBPA point mutations in hematological malignancies. Leukemia.

[218] Zhang P., Iwasaki-Arai J., Iwasaki H., Fenyus M.L., Dayaram T., Owens B.M., Shigematsu H., Levantini E., Huettner C.S., Lekstrom-Himes J.A. (2004). Enhancement of hematopoietic stem cell repopulating capacity and self-renewal in the absence of the transcription factor C/EBP alpha. Immunity.

[219] Braun T.P., Okhovat M., Coblentz C., Carratt S.A., Foley A., Schonrock Z., Smith B.M., Nevonen K., Davis B., Garcia B. (2019). Myeloid lineage enhancers drive oncogene synergy in CEBPA/CSF3R mutant acute myeloid leukemia. Nat. Commun..

[220] Ohlsson E., Hasemann M.S., Willer A., Lauridsen F.K., Rapin N., Jendholm J., Porse B.T. (2014). Initiation of MLL-rearranged AML is dependent on C/EBPα. J. Exp. Med..

[221] Collins C., Wang J., Miao H., Bronstein J., Nawer H., Xu T., Figueroa M., Muntean A.G., Hess J.L. (2014). C/EBPα is an essential collaborator in Hoxa9/Meis1-mediated leukemogenesis. Proc. Natl. Acad. Sci. USA.

[222] Takei H., Kobayashi S.S. (2019). Targeting transcription factors in acute myeloid leukemia. Int. J. Hematol..

[223] Okuda T., Nishimura M., Nakao M., Fujita Y. (2001). RUNX1/AML1: A central player in hematopoiesis. Int. J. Hematol..

[224] Growney J.D., Shigematsu H., Li Z., Lee B.H., Adelsperger J., Rowan R., Curley D.P., Kutok J.L., Akashi K., Williams I.R. (2005). Loss of Runx1 perturbs adult hematopoiesis and is associated with a myeloproliferative phenotype. Blood.

[225] Schnittger S., Dicker F., Kern W., Wendland N., Sundermann J., Alpermann T., Haferlach C., Haferlach T. (2011). RUNX1 mutations are frequent in de novo AML with noncomplex karyotype and confer an unfavorable prognosis. Blood.

[226] Chin D.W., Watanabe-Okochi N., Wang C.Q., Tergaonkar V., Osato M. (2015). Mouse models for core binding factor leukemia. Leukemia.

[227] Bera R., Chiu M.C., Huang Y.J., Lin T.H., Kuo M.C., Shih L.Y. (2019). RUNX1 mutations promote leukemogenesis of myeloid malignancies in ASXL1-mutated leukemia. J. Hematol. Oncol..

[228] Goyama S., Schibler J., Cunningham L., Zhang Y., Rao Y., Nishimoto N., Nakagawa M., Olsson A., Wunderlich M., Link K.A. (2013). Transcription factor RUNX1 promotes survival of acute myeloid leukemia cells. J. Clin. Investig..

[229] Duffy M.J., O’Grady S., Tang M., Crown J. (2021). MYC as a target for cancer treatment. Cancer Treat. Rev..

[230] Chen H., Liu H., Qing G. (2018). Targeting oncogenic Myc as a strategy for cancer treatment. Signal. Transduct. Target. Ther..

[231] Delgado M.D., León J. (2010). Myc roles in hematopoiesis and leukemia. Genes Cancer.

[232] Astolfi A., Fiore M., Melchionda F., Indio V., Bertuccio S.N., Pession A. (2019). BCOR involvement in cancer. Epigenomics.

[233] Grossmann V., Tiacci E., Holmes A.B., Kohlmann A., Martelli M.P., Kern W., Spanhol-Rosseto A., Klein H.U., Dugas M., Schindela S. (2011). Whole-exome sequencing identifies somatic mutations of BCOR in acute myeloid leukemia with normal karyotype. Blood.

[234] Schmidt C.R., Achille N.J., Kuntimaddi A., Boulton A.M., Leach B.I., Zhang S., Zeleznik-Le N.J., Bushweller J.H. (2020). BCOR Binding to MLL-AF9 Is Essential for Leukemia via Altered EYA1, SIX, and MYC Activity. Blood Cancer Discov..

[235] McNerney M.E., Brown C.D., Wang X., Bartom E.T., Karmakar S., Bandlamudi C., Yu S., Ko J., Sandall B.P., Stricker T. (2013). CUX1 is a haploinsufficient tumor suppressor gene on chromosome 7 frequently inactivated in acute myeloid leukemia. Blood.

[236] Aly M., Ramdzan Z.M., Nagata Y., Balasubramanian S.K., Hosono N., Makishima H., Visconte V., Kuzmanovic T., Adema V., Nazha A. (2019). Distinct clinical and biological implications of CUX1 in myeloid neoplasms. Blood Adv..

[237] An N., Khan S., Imgruet M.K., Gurbuxani S.K., Konecki S.N., Burgess M.R., McNerney M.E. (2018). Gene dosage effect of CUX1 in a murine model disrupts HSC homeostasis and controls the severity and mortality of MDS. Blood.

[238] Stieglitz E., Troup C.B., Gelston L.C., Haliburton J., Chow E.D., Yu K.B., Akutagawa J., Taylor-Weiner A.N., Liu Y.L., Wang Y.-D. (2015). Subclonal mutations in SETBP1 confer a poor prognosis in juvenile myelomonocytic leukemia. Blood.

[239] Thol F., Suchanek K.J., Koenecke C., Stadler M., Platzbecker U., Thiede C., Schroeder T., Kobbe G., Kade S., Löffeld P. (2013). SETBP1 mutation analysis in 944 patients with MDS and AML. Leukemia.

[240] Makishima H., Yoshida K., Nguyen N., Przychodzen B., Sanada M., Okuno Y., Ng K.P., Gudmundsson K.O., Vishwakarma B.A., Jerez A. (2013). Somatic SETBP1 mutations in myeloid malignancies. Nat. Genet..

[241] Lower K.M., Turner G., Kerr B.A., Mathews K.D., Shaw M.A., Gedeon A.K., Schelley S., Hoyme H.E., White S.M., Delatycki M.B. (2002). Mutations in PHF6 are associated with Börjeson-Forssman-Lehmann syndrome. Nat. Genet..

[242] Van Vlierberghe P., Patel J., Abdel-Wahab O., Lobry C., Hedvat C.V., Balbin M., Nicolas C., Payer A.R., Fernandez H.F., Tallman M.S. (2011). PHF6 mutations in adult acute myeloid leukemia. Leukemia.

[243] Patel J.P., Gonen M., Figueroa M.E., Fernandez H., Sun Z., Racevskis J., Van Vlierberghe P., Dolgalev I., Thomas S., Aminova O. (2012). Prognostic relevance of integrated genetic profiling in acute myeloid leukemia. N. Engl. J. Med..

[244] Todd M.A., Ivanochko D., Picketts D.J. (2015). PHF6 Degrees of Separation: The Multifaceted Roles of a Chromatin Adaptor Protein. Genes.

[245] King-Underwood L., Little S., Baker M., Clutterbuck R., Delassus S., Enver T., Lebozer C., Min T., Moore A., Schedl A. (2005). Wt1 is not essential for hematopoiesis in the mouse. Leuk. Res..

[246] Chau Y.Y., Brownstein D., Mjoseng H., Lee W.C., Buza-Vidas N., Nerlov C., Jacobsen S.E., Perry P., Berry R., Thornburn A. (2011). Acute multiple organ failure in adult mice deleted for the developmental regulator Wt1. PLoS Genet..

[247] Miwa H., Beran M., Saunders G.F. (1992). Expression of the Wilms’ tumor gene (WT1) in human leukemias. Leukemia.

[248] King-Underwood L., Renshaw J., Pritchard-Jones K. (1996). Mutations in the Wilms’ tumor gene WT1 in leukemias. Blood.

[249] Menssen H.D., Renkl H.J., Rodeck U., Maurer J., Notter M., Schwartz S., Reinhardt R., Thiel E. (1995). Presence of Wilms’ tumor gene (wt1) transcripts and the WT1 nuclear protein in the majority of human acute leukemias. Leukemia.

[250] Becker H., Marcucci G., Maharry K., Radmacher M.D., Mrozek K., Margeson D., Whitman S.P., Paschka P., Holland K.B., Schwind S. (2010). Mutations of the Wilms tumor 1 gene (WT1) in older patients with primary cytogenetically normal acute myeloid leukemia: A Cancer and Leukemia Group B study. Blood.

[251] Pritchard-Jones K., Fleming S., Davidson D., Bickmore W., Porteous D., Gosden C., Bard J., Buckler A., Pelletier J., Housman D. (1990). The candidate Wilms’ tumour gene is involved in genitourinary development. Nature.

[252] Rampal R., Alkalin A., Madzo J., Vasanthakumar A., Pronier E., Patel J., Li Y., Ahn J., Abdel-Wahab O., Shih A. (2014). DNA hydroxymethylation profiling reveals that WT1 mutations result in loss of TET2 function in acute myeloid leukemia. Cell Rep..

[253] Rampal R., Figueroa M.E. (2016). Wilms tumor 1 mutations in the pathogenesis of acute myeloid leukemia. Haematologica.

[254] Barbosa K., Li S., Adams P.D., Deshpande A.J. (2019). The role of TP53 in acute myeloid leukemia: Challenges and opportunities. Genes Chromosomes Cancer.

[255] Robles A.I., Harris C.C. (2010). Clinical outcomes and correlates of TP53 mutations and cancer. Cold Spring Harb. Perspect. Biol..

[256] Liu Y., Elf S.E., Miyata Y., Sashida G., Liu Y., Huang G., Di Giandomenico S., Lee J.M., Deblasio A., Menendez S. (2009). p53 regulates hematopoietic stem cell quiescence. Cell Stem Cell.

[257] Chen J., Ellison F.M., Keyvanfar K., Omokaro S.O., Desierto M.J., Eckhaus M.A., Young N.S. (2008). Enrichment of hematopoietic stem cells with SLAM and LSK markers for the detection of hematopoietic stem cell function in normal and Trp53 null mice. Exp. Hematol..

[258] TeKippe M., Harrison D.E., Chen J. (2003). Expansion of hematopoietic stem cell phenotype and activity in Trp53-null mice. Exp. Hematol..

[259] Pant V., Quintas-Cardama A., Lozano G. (2012). The p53 pathway in hematopoiesis: Lessons from mouse models, implications for humans. Blood.

[260] Liu G., Parant J.M., Lang G., Chau P., Chavez-Reyes A., El-Naggar A.K., Multani A., Chang S., Lozano G. (2004). Chromosome stability, in the absence of apoptosis, is critical for suppression of tumorigenesis in Trp53 mutant mice. Nat. Genet..

[261] Lang G.A., Iwakuma T., Suh Y.A., Liu G., Rao V.A., Parant J.M., Valentin-Vega Y.A., Terzian T., Caldwell L.C., Strong L.C. (2004). Gain of function of a p53 hot spot mutation in a mouse model of Li-Fraumeni syndrome. Cell.

[262] Olive K.P., Tuveson D.A., Ruhe Z.C., Yin B., Willis N.A., Bronson R.T., Crowley D., Jacks T. (2004). Mutant p53 gain of function in two mouse models of Li-Fraumeni syndrome. Cell.

[263] Basova P., Pospisil V., Savvulidi F., Burda P., Vargova K., Stanek L., Dluhosova M., Kuzmova E., Jonasova A., Steidl U. (2014). Aggressive acute myeloid leukemia in PU.1/p53 double-mutant mice. Oncogene.

[264] Jyotsana N., Heuser M. (2018). Exploiting differential RNA splicing patterns: A potential new group of therapeutic targets in cancer. Expert Opin. Ther. Targets.

[265] Papaemmanuil E., Gerstung M., Malcovati L., Tauro S., Gundem G., Van Loo P., Yoon C.J., Ellis P., Wedge D.C., Pellagatti A. (2013). Clinical and biological implications of driver mutations in myelodysplastic syndromes. Blood.

[266] Haferlach T., Nagata Y., Grossmann V., Okuno Y., Bacher U., Nagae G., Schnittger S., Sanada M., Kon A., Alpermann T. (2014). Landscape of genetic lesions in 944 patients with myelodysplastic syndromes. Leukemia.

[267] Steensma D.P., Bejar R., Jaiswal S., Lindsley R.C., Sekeres M.A., Hasserjian R.P., Ebert B.L. (2015). Clonal hematopoiesis of indeterminate potential and its distinction from myelodysplastic syndromes. Blood.

[268] Bamopoulos S.A., Batcha A.M.N., Jurinovic V., Rothenberg-Thurley M., Janke H., Ksienzyk B., Philippou-Massier J., Graf A., Krebs S., Blum H. (2020). Clinical presentation and differential splicing of SRSF2, U2AF1 and SF3B1 mutations in patients with acute myeloid leukemia. Leukemia.

[269] Lee S.C., Dvinge H., Kim E., Cho H., Micol J.B., Chung Y.R., Durham B.H., Yoshimi A., Kim Y.J., Thomas M. (2016). Modulation of splicing catalysis for therapeutic targeting of leukemia with mutations in genes encoding spliceosomal proteins. Nat. Med..

[270] Yoshimi A., Lin K.-T., Wiseman D.H., Rahman M.A., Pastore A., Wang B., Lee S.C.-W., Micol J.-B., Zhang X.J., de Botton S. (2019). Coordinated alterations in RNA splicing and epigenetic regulation drive leukaemogenesis. Nature.

[271] Huang Y.-J., Yan M., Kim E., Davis A.G., Shima T., Miyauchi S., Fu X.-D., Abdel-Wahab O., Zhang D.-E. (2017). RUNX1 Deficiency and SRSF2 Mutation Cooperate to Promote Myelodysplastic Syndrome Development. Blood.

[272] Wang E., Aifantis I. (2020). RNA Splicing and Cancer. Trends Cancer.

[273] Heimbruch K.E., Meyer A.E., Agrawal P., Viny A.D., Rao S. (2021). A cohesive look at leukemogenesis: The cohesin complex and other driving mutations in AML. Neoplasia.

[274] Cuartero S., Innes A.J., Merkenschlager M. (2019). Towards a Better Understanding of Cohesin Mutations in AML. Front. Oncol..

[275] Zhu Z., Wang X. (2019). Roles of cohesin in chromosome architecture and gene expression. Semin. Cell Dev. Biol..

[276] Han C., Gao X., Li Y., Zhang J., Yang E., Zhang L., Yu L. (2021). Characteristics of Cohesin Mutation in Acute Myeloid Leukemia and Its Clinical Significance. Expert Opin. Ther. Targets.

[277] Fang C., Rao S., Crispino J.D., Ntziachristos P. (2020). Determinants and role of chromatin organization in acute leukemia. Leukemia.

[278] Thol F., Bollin R., Gehlhaar M., Walter C., Dugas M., Suchanek K.J., Kirchner A., Huang L., Chaturvedi A., Wichmann M. (2014). Mutations in the cohesin complex in acute myeloid leukemia: Clinical and prognostic implications. Blood.

[279] Welch J.S., Ley T.J., Link D.C., Miller C.A., Larson D.E., Koboldt D.C., Wartman L.D., Lamprecht T.L., Liu F., Xia J. (2012). The origin and evolution of mutations in acute myeloid leukemia. Cell.

[280] Mintzas K., Heuser M. (2019). Emerging strategies to target the dysfunctional cohesin complex in cancer. Expert Opin. Ther. Targets.

[281] Mullenders J., Aranda-Orgilles B., Lhoumaud P., Keller M., Pae J., Wang K., Kayembe C., Rocha P.P., Raviram R., Gong Y. (2015). Cohesin loss alters adult hematopoietic stem cell homeostasis, leading to myeloproliferative neoplasms. J. Exp. Med..

[282] Viny A.D., Bowman R.L., Liu Y., Lavallee V.P., Eisman S.E., Xiao W., Durham B.H., Navitski A., Park J., Braunstein S. (2019). Cohesin Members Stag1 and Stag2 Display Distinct Roles in Chromatin Accessibility and Topological Control of HSC Self-Renewal and Differentiation. Cell Stem Cell.

[283] Ochi Y., Kon A., Sakata T., Nakagawa M.M., Nakazawa N., Kakuta M., Kataoka K., Koseki H., Nakayama M., Morishita D. (2020). Combined Cohesin-RUNX1 Deficiency Synergistically Perturbs Chromatin Looping and Causes Myelodysplastic Syndromes. Cancer Discov..

[284] Tsai C.H., Hou H.A., Tang J.L., Kuo Y.Y., Chiu Y.C., Lin C.C., Liu C.Y., Tseng M.H., Lin T.Y., Liu M.C. (2017). Prognostic impacts and dynamic changes of cohesin complex gene mutations in de novo acute myeloid leukemia. Blood Cancer J..

