# Supplementary material for: Mouse Models of Frequently Mutated Genes in Acute Myeloid Leukemia
Source: Cancers (Basel). 2021 Dec 8;13(24):6192. doi: 10.3390/cancers13246192 (PMC8699817; doi:10.3390/cancers13246192)
Supplement: Supplementary file 1 [file cancers-13-06192-s001.zip › cancers-1423779-supplementary.pdf]

cancers
MDPI

*Review*

# Mouse Models of Frequently Mutated Genes in Acute Myeloid Leukemia

**Sagarajit Mohanty and Michael Heuser**

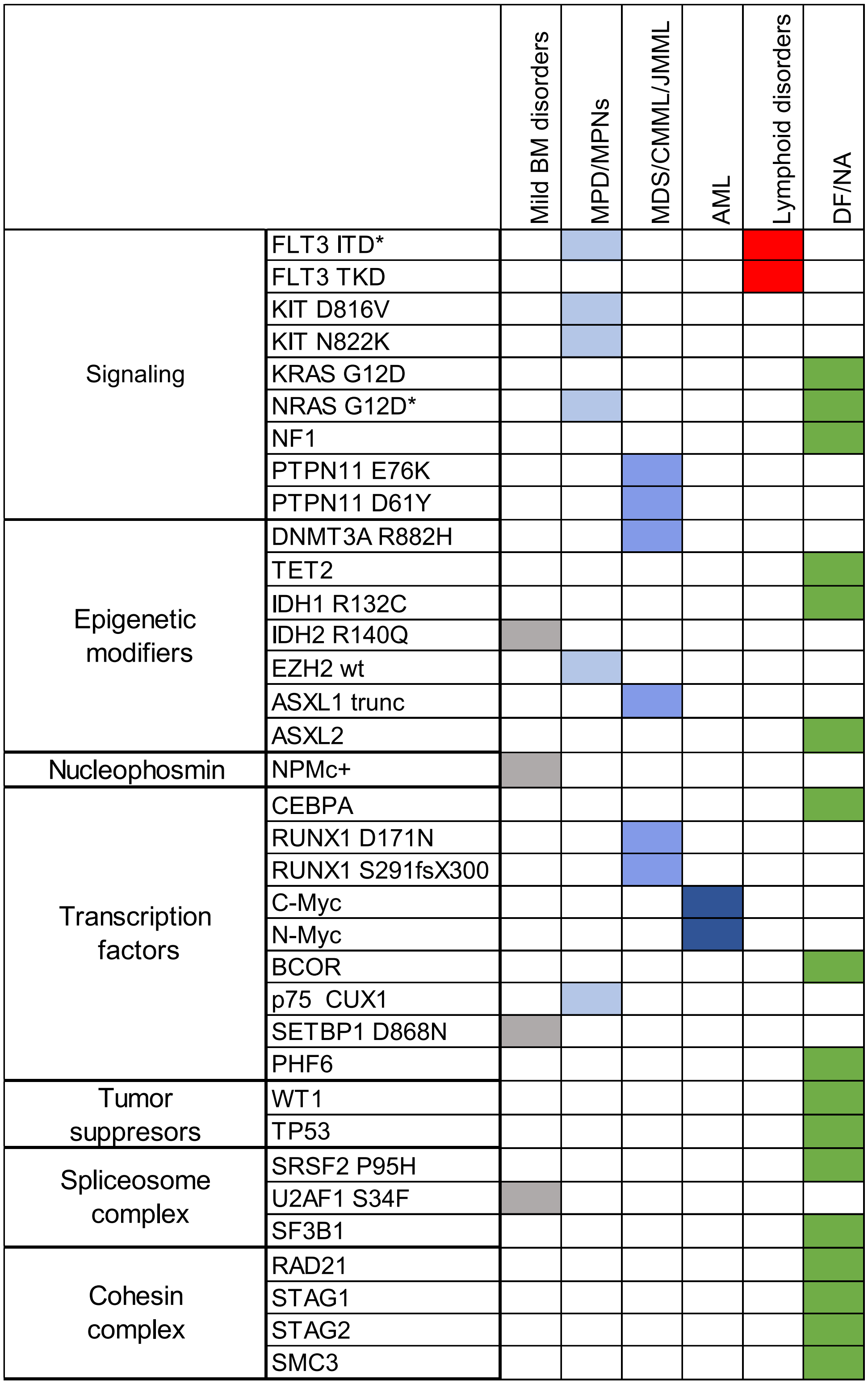


**Figure S1.** Disease induction by frequently mutated genes in transgenic/overexpression mouse models. We have divided the figure into 6 different categories. The first category includes any mild bone marrow disease or splenomegaly but not any defined disease. Myeloproliferative disease (MPD)/Myeloproliferative Neoplasms (MPN) is included in the second category. Myelodysplastic syndromes (MDS), chronic myelomonocytic leukemia (CMML), and juvenile myelomonocytic leukemia (JMML) are listed under the third category. The fourth category includes mutations that cause AML induction in mice. The fifth category includes mutations that cause any kind of lymphoid disorders. The sixth category contains

the genetic mutations that either do not cause any disease or arnot explained yet. Mild BM disorders-Grey, Myeloproliferative disease (MPD)-Light Blue, Myeloproliferative Neoplasms (MPNs)-Light Blue, Myelodysplastic syndromes (MDS)-Blue, Chronic myelomonocytic leukemia (CMML)-Blue, Juvenile myelomonocytic leukemia (JMML)-Blue, Acute myeloid leukemia (AML)-Dark blue, Lymphoid disorders-Red, Disease free (DF)-Green, Not available (NA)-Green, Wildtype (wt), Truncated (trunc).

| | | Mild BM disorders | MPD/MPN | MDS/CMML/JMML | AML | Lymphoid disorders | DF/NA |
|---|---|---|---|---|---|---|---|
| Signaling | FLT3 ITD* | | | | | | |
| | FLT3 D835Y | | | | | | |
| | KIT | | | | | | |
| | KRAS G12D | | | | | | |
| | NRAS G12D | | | | | | |
| | NF1 | | | | | | |
| | PTPN11 D61Y | | | | | | |
| | PTPN11 E76K | | | | | | |
| Epigenetic modifier | DNMT3A R878H | | | | | | |
| | TET2 | | | | | | |
| | IDH1 R132H | | | | | | |
| | IDH2 R140Q | | | | | | |
| | EZH2 | | | | | | |
| | ASXL1 G643fs* | | | | | | |
| | ASXL2 | | | | | | |
| Nucleophosmin | NPM1 | | | | | | |
| Transcription factors | CEBPα K313KK/ Lp30 | | | | | | |
| | RUNX1 | | | | | | |
| | MYC | | | | | | |
| | BCOR ΔE4/y | | | | | | |
| | BCOR ΔE9-10/y | | | | | | |
| | CUX1 | | | | | | |
| | SETBP1 | | | | | | |
| | PHF6 | | | | | | |
| Tumor suppresors | WT1 R394W | | | | | | |
| | TP53 R172H | | | | | | |
| | TP53 R248Q | | | | | | |
| Splicesome complex | SRSF2 P95H | | | | | | |
| | U2AF1 S34F | | | | | | |
| | SF3B1 K700E* | | | | | | |
| Cohesin complex | RAD21 | | | | | | |
| | STAG1 | | | | | | |
| | STAG2 | | | | | | |
| | SMC3 | | | | | | |

**Figure S2.** Disease induction by frequently mutated genes in knockin mouse models. Knockin mouse models show mutations in the targeted genomic locus and transgenic/overexpression mouse models are characterized by random integrations of the target gene in the genome. Mild BM disorders-Grey, Myeloproliferative disease (MPD)-Light Blue, Myeloproliferative Neoplasms (MPNs)-Light Blue, Myelodysplastic syndromes (MDS)-Blue, Chronic myelomonocytic leukemia (CMML)-Blue, Juvenile myelomonocytic leukemia (JMML)-Blue, Acute myeloid leukemia (AML)-Dark blue, Lymphoid disorders-Red, Disease free (DF)-Green, Not available (NA)-Green, CEBPA C-terminal mutation K313 duplication: K313KK N-terminal mutation (Lp30 allele).

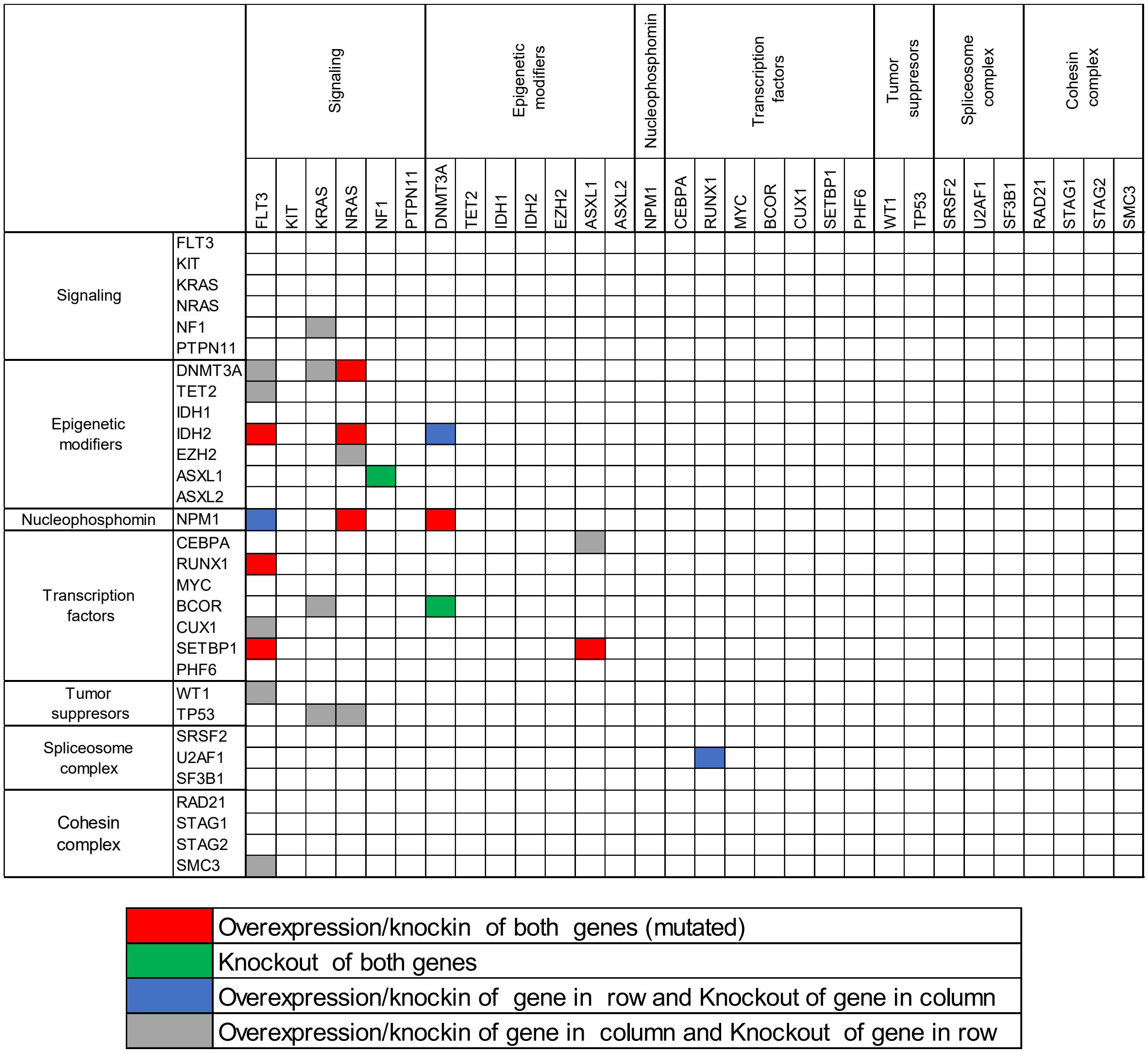


**Figure S3.** Map of genes that functionally cooperate in vivo. This matrix illustrates that mutations in signaling genes are the most frequent cooperation partners of mutations in the other functional pathways. It also shows that signaling genes almost always function by overexpression/knockin (red or grey color), while the cooperation partners in the other pathways are either activated (red) or inactivated (grey).
